# Reduced fractional anisotropy in bipolar disorder *v.* major depressive disorder independent of current symptoms

**DOI:** 10.1017/S0033291722001490

**Published:** 2023-07

**Authors:** Katharina Thiel, Susanne Meinert, Alexandra Winter, Hannah Lemke, Lena Waltemate, Fabian Breuer, Marius Gruber, Ramona Leenings, Lucia Wüste, Kathrin Rüb, Julia-Katharina Pfarr, Frederike Stein, Katharina Brosch, Tina Meller, Kai Gustav Ringwald, Igor Nenadić, Axel Krug, Jonathan Repple, Nils Opel, Katharina Koch, Elisabeth J. Leehr, Jochen Bauer, Dominik Grotegerd, Tim Hahn, Tilo Kircher, Udo Dannlowski

**Affiliations:** 1 Institute for Translational Psychiatry, University of Münster, Münster, Germany; 2 Institute of Translational Neuroscience, University of Münster, Münster, Germany; 3 Department of Psychiatry and Psychotherapy, University of Marburg, Marburg, Germany; 4 Department of Psychiatry and Psychotherapy, University Hospital Bonn, Bonn, Germany; 5 Department of Clinical Radiology, University of Muenster, Muenster, Germany

**Keywords:** Bipolar disorder, diffusion tensor imaging, major depressive disorder, mood state, trait, white matter microstructure

## Abstract

**Background:**

Patients with bipolar disorder (BD) show reduced fractional anisotropy (FA) compared to patients with major depressive disorder (MDD). Little is known about whether these differences are mood state-independent or influenced by acute symptom severity. Therefore, the aim of this study was (1) to replicate abnormalities in white matter microstructure in BD *v.* MDD and (2) to investigate whether these vary across depressed, euthymic, and manic mood.

**Methods:**

In this cross-sectional diffusion tensor imaging study, *n* = 136 patients with BD were compared to age- and sex-matched MDD patients and healthy controls (HC) (*n* = 136 each). Differences in FA were investigated using tract-based spatial statistics. Using interaction models, the influence of acute symptom severity and mood state on the differences between patient groups were tested.

**Results:**

Analyses revealed a main effect of diagnosis on FA across all three groups (*p_tfce-FWE_* = 0.003). BD patients showed reduced FA compared to both MDD (*p_tfce-FWE_* = 0.005) and HC (*p_tfce-FWE_* < 0.001) in large bilateral clusters. These consisted of several white matter tracts previously described in the literature, including commissural, association, and projection tracts. There were no significant interaction effects between diagnosis and symptom severity or mood state (all *p_tfce-FWE_* > 0.704).

**Conclusions:**

Results indicated that the difference between BD and MDD was independent of depressive and manic symptom severity and mood state. Disruptions in white matter microstructure in BD might be a trait effect of the disorder. The potential of FA values to be used as a biomarker to differentiate BD from MDD should be further addressed in future studies using longitudinal designs.

## Introduction

Bipolar disorder (BD) can be distinguished from major depressive disorder (MDD) by the presence of manic or hypomanic episodes. However, they are very similar in depressive psychopathology, as the same diagnostic criteria for depressive episodes apply to both disorders. In addition, the majority of BD patients present clinically primarily with depressive symptoms without reporting previous (hypo)manic episodes. This results in up to 70% of BD patients being initially misdiagnosed as MDD and an average of 5–10 years passing before the correct diagnosis is made (Berk et al., 2007; Grande, Berk, Birmaher, & Vieta, 2016; Hirschfeld, Lewis, & Vornik, 2003). This may cause a delay in the selection of effective psychotherapeutic and pharmacological interventions, resulting in poor disease course and prognosis as well as higher healthcare costs (De Almeida & Phillips, 2013; Hirschfeld et al., 2003; Phillips & Kupfer, 2013). Therefore, identifying neurobiological markers of BD that aid in differential diagnosis between BD and MDD is of high clinical and scientific significance and has been subject to recent neuroimaging research (Han, De Berardis, Fornaro, & Kim, 2019; Phillips & Swartz, 2014; Versace et al., 2010). One promising research subject is the microstructure of white matter (WM) (De Almeida & Phillips, 2013; Phillips & Kupfer, 2013). It can be studied by the magnetic resonance imaging (MRI) derived technique of diffusion tensor imaging (DTI), which quantifies water diffusion and the degree of its directionality in neuronal tissue. The most commonly examined measure in DTI studies is fractional anisotropy (FA), which is interpreted as a measure of WM integrity, myelination, coherence, and density of fiber bundling (Jones, Knösche, & Turner, 2013; Soares, Marques, Alves, & Sousa, 2013).

Alterations in WM microstructure have been shown to be present in both BD and MDD when compared to healthy controls (HC). Findings point toward a reduction of FA in both disorders, mainly in frontal and temporal WM tracts connecting the prefrontal cortex and anterior limbic structures (Chen et al., 2016; Duarte, De Araújo e Silva, Goldani, Massuda, & Gama, 2016; Phillips & Swartz, 2014; van Velzen et al., 2020). A recent meta-analysis on WM abnormalities in BD and MDD patients compared the effect sizes between these disorders and found a greater reduction in FA in BD than in MDD compared to HC in the left posterior cingulum (Wise et al., 2016). However, included studies contrasted both patient groups separately with HC, so this meta-analysis only compares these disorders indirectly. To date, DTI studies directly comparing MDD and BD have been rare, although they are crucial to identify changes in WM microstructure that clearly distinguish both disorders (De Almeida & Phillips, 2013; Han et al., 2019; Phillips & Swartz, 2014). Consistent with the aforementioned meta-analysis (Wise et al., 2016), most existing findings suggest that BD patients show reduced FA in WM compared to MDD patients. Affected tracts include the body and genu of the corpus callosum (Masuda et al., 2020; Matsuoka et al., 2017; Repple et al., 2017), cingulum bundles (Benedetti et al., 2011a; Repple et al., 2017), uncinate fasciculi (Benedetti et al., 2011a; Deng et al., 2018), corticospinal tracts (Metin, Altuglu, Metin, & Tarhan, 2020; Repple et al., 2017), and superior longitudinal fasciculi (Repple et al., 2017; Versace et al., 2010), which are thought to form parts of the fronto-temporal system involved in the processing and regulation of emotion (Manelis et al., 2021).

A number of researchers point out the importance of considering different mood states when comparing the two disorders on a neurobiological level to identify differences in neural structures that persist across all disease phases (Dvorak et al., 2019; Han et al., 2019; Phillips, 2019; Sacchet, Livermore, Iglesias, Glover, & Gotlib, 2015). However, the majority of studies limited their sample to patients of one mood state, with most of these studies examining currently depressed patients (Deng et al., 2018; Lan et al., 2020; Repple et al., 2017; Vai et al., 2020; Versace et al., 2010) and only one looking at euthymic patients (Masuda et al., 2020). Inclusion of euthymic as well as currently (hypo)manic patients is essential to determine whether these are transient differences associated with acute symptoms or stable, mood state-independent changes that can be regarded as trait differences and may help to distinguish both disorders (Benedetti et al., 2011b; Dvorak et al., 2019; Masuda et al., 2020; Phillips, 2019). Two DTI studies that directly compared BD patients with different mood states to HC suggest that WM microstructure varies with mood state, with currently depressed patients showing widely distributed reductions in FA when compared to HC that are less widespread or even absent in the manic and euthymic states (Magioncalda et al., 2016; Zanetti et al., 2009). In contrast, Cui et al. (2020) reported no significant differences in FA between manic and depressed BD patients. To the best of our knowledge, only one DTI study examined differences in WM microstructure between BD and MDD while accounting for current mood (Matsuoka et al., 2017). In a sample including both currently depressed and euthymic – but no (hypo)manic – patients, this study found reduced FA in the anterior part of the corpus callosum in BD compared to MDD patients. Results remained significant after including the severity of depressive symptoms as a covariate (Matsuoka et al., 2017).

Thus, this study aimed to investigate differences in WM microstructure, particularly in FA, between BD and MDD patients and HC in a relatively large sample including patients in euthymic, depressed, and (hypo)manic mood state, and to explore whether these differences vary as a function of current mood. First, we expect alterations in WM microstructure, particularly a reduction of FA, in both BD and MDD patients compared to HC in fronto-temporal WM tracts (hypothesis 1). Second, we expect even more severe impairments of WM microstructure in these tracts in BD as compared to MDD patients (hypothesis 2). Given the paucity of findings, we have no specific hypothesis regarding the role of patients' current mood. We explore whether differences between BD and MDD patients vary as a function of their current mood, which we capture in two ways: First, using a dimensional approach, via current depressive and manic symptom severity, and second, using a categorical classification of euthymic, depressed, and manic mood states (hypothesis 3).

## Materials and methods

### Participants

The present study comprised data from the FOR2107-cohort which were collected at two scanning sites – the University of Marburg and the University of Münster [see earlier work for the general description of the study (Kircher et al., 2019) and the MRI quality assurance protocol (Vogelbacher et al., 2018)].

The FOR2107 study was approved by the Ethics Committees of the Medical Faculties, University of Marburg (AZ: 07/14) and University of Münster (2014-422-b-S). The authors assert that all procedures contributing to this work comply with the ethical standards of the relevant national and institutional committees on human experimentation and with the Helsinki Declaration of 1975, as revised in 2008. All subjects provided written informed consent prior to examination and received financial compensation for participation. Participants were recruited in psychiatric hospitals or via newspaper advertisements.

See online Supplementary material 1 for detailed information on inclusion and exclusion criteria and the selection of the sample. In total, 408 participants were included in the study. *N* = 136 individuals with BD (*n* = 75 female, *M_age_* = 41.17, s.d.*_age_* = 11.98, *n* = 73 BD type I, *n* = 63 BD type II) were selected. The same number of individuals with MDD as well as HC were matched to these subjects regarding age, sex, and site (MDD: *n* = 79 female, *M_age_* = 41.59, s.d.*_age_* = 12.43, HC: *n* = 77 female, *M_age_* = 42.26, s.d.*_age_* = 12.92), using the MatchIt package in R (2020, Version 4.0.1) (Ho, Imai, King, & Stuart, 2011). Table 1 provides detailed information on sociodemographic and clinical characteristics of the sample, Table 2 shows the latter separately for the euthymic, depressed, and (hypo)manic states. All participants underwent the Structured Clinical Interview for DSM-IV (SCID-I; Wittchen, Wunderlich, Gruschwitz, & Zaudig, 1997) to verify lifetime psychiatric diagnoses or the lack thereof. Remission status and current affective state were determined based on DSM-IV criteria (online Supplementary Table S2). Any lifetime psychiatric disorder according to the SCID-I as well as any intake of psychotropic medication resulted in exclusion from the study for HC. The 21-item Hamilton Depression Rating Scale (HDRS) (Hamilton, 1960) and the Young Mania Rating Scale (YMRS) (Young, Biggs, Ziegler, & Meyer, 1978) were employed to assess the presence and severity of current depressive and manic symptoms, respectively. The Global Assessment of Functioning was used to rate the participants' overall functioning (Saß & Wittchen, 2003). By using an established strategy as described earlier (Hassel et al., 2008; Redlich et al., 2014), we calculated the Medication Load Index (MedIndex) for each patient, a composite measure of individual total medication load reflecting the number and daily dose of all psychopharmacological medication (online Supplementary material 2).

**Table 1.** Demographic and clinical characteristics of the study sample

	BD (*n* = 136)	MDD (*n* = 136)	Tests for the two patient groups		HC (*n* = 136)	Tests for all three groups	
			Test statistic	*p* value		Test statistic	*p* value
Sociodemographic characteristics							
Age, M ± s.d.	41.17 ± 11.98	41.59 ± 12.43	0.283^1^	0.777	42.26 ± 12.92	0.264^4^	0.768
Sex (male/female)	61/75	57/79	0.239^2^	0.625	59/77	0.239^2^	0.887
Years of education^5^, M ± s.d.	14.02 ± 2.78	13.41 ± 2.87	−1.758^1^	0.080	14.26 ± 2.76	3.33^4^	0.037
Site (Marburg/Münster)	63/73	63/73	0^2^	1.000	72/64	1.59^2^	0.452
Questionnaires							
YMRS^5^, M ± s.d.	3.73 ± 5.53	1.39 ± 2.33	−4.536^3^	<0.001	0.49 ± 1.14	30.59^4^	<0.001
HDRS^5^, M ± s.d.	7.51 ± 6.52	8.64 ± 7.54	1.321^1^	0.187	1.02 ± 1.48	67.984^4^	<0.001
GAF^5^, M ± s.d.	62.53 ± 12.46	65.51 ± 16.0	1.697^3^	0.091	90.43 ± 7.33	201.382^4^	<0.001
Clinical characteristics							
Number of lifetime depressive episodes^5^, M ± s.d.	7.79 ± 7.70	3.94 ± 4.56	−4.866^3^	<0.001	n/a	n/a	n/a
Lifetime duration of depressive episodes (months)^5^, M ± s.d.	50.79 ± 71.35	52.38 ± 85.15	0.151^1^	0.880	n/a	n/a	n/a
Number of lifetime (hypo-)manic episodes^5^, M ± s.d.	5.98 ± 8.22	n/a	n/a	n/a	n/a	n/a	n/a
Lifetime duration of (hypo-)manic episodes (months)^5^, M ± s.d.	18.92 ± 36.81	n/a	n/a	n/a	n/a	n/a	n/a
Number of lifetime hospitalizations^5^, M ± s.d.	3.73 ± 3.16	1.78 ± 2.16	−5.890^3^	<0.001	n/a	n/a	n/a
Lifetime duration of hospitalization (weeks)^5^, M ± s.d.	32.70 ± 33.52	12.35 ± 15.25	−6.311^3^	<0.001	n/a	n/a	n/a
Age of onset (years), M ± s.d.	24.09 ± 11.11	29.14 ± 12.75	3.456^3^	0.001	n/a	n/a	n/a
Comorbid diagnoses (yes/no)	54/82	56/80	0.061^2^	0.805	n/a	n/a	n/a
Current inpatient treatment (yes/no)	39/93	50/86	1.57^2^	0.210	n/a	n/a	n/a
Medication load index^6^, M ± s.d.	2.57 ± 2.04	1.65 ± 1.70	−4.041^1^	<0.001	n/a	n/a	n/a
Current lithium intake (yes/no)^6^	38/98	5/131	30.08^2^	<0.001	n/a	n/a	n/a
BD subtype (BD I/BD II)	73/63	n/a	n/a	n/a	n/a	n/a	n/a

BD, bipolar disorder; GAF, Global Assessment of Functioning; HC, healthy controls; HDRS, Hamilton Depression Rating Scale; MDD, major depressive disorder; s.d., standard deviation; YMRS, Young Mania Rating Scale; n, number; n/a, not applicable.

*Note:* ^1^Two-sample *t* test assuming equal variance, ^2^Pearson *χ^2^* test, ^3^two-sample *t* test assuming unequal variance, ^4^one-way analysis of variance (ANOVA) *F*-test, ^5^not all participants provided the necessary information, *N*_min_ = 112 BD, *N*_min_ = 108 MDD, see Table 2 for detailed information, ^6^see online Supplementary Table S1 for detailed information on medication intake.

**Table 2.** Clinical characteristics of patient subgroups

	BD euthymic^a^ (*n* = 39)	BD depressed^b^ (*n* = 53)	BD (hypo-)manic^c^ (*n* = 30)	MDD euthymic^d^ (*n* = 38)	MDD depressed^e^ (*n* = 98)	Group comparison^1^
Questionnaires						
HDRS, M ± s.d.	3.54 ± 3.81	12.25 ± 6.52	4.31 ± 3.81 (*n* = 29)	2.16 ± 2.43	11.19 ± 7.34 (*n* = 97)	*F* = 32.31, *p* < 0.001^2^ (ab, ae, bc, bd, cd, ce, de)
YMRS, M ± s.d.	1.15 ± 2.03	3.42 ± 4.51	8.14 ± 8.24 (*n* = 29)	1.18 ± 1.72	1.47 ± 2.53 (*n* = 97)	*F* = 19.68, *p* < 0.001^2^ (ab, ac, bc, bd, be, cd, ce)
GAF, M ± s.d.	72.59 ± 12.54	58.56 ± 8.79 (*n* = 52)	57.52 ± 11.81 (*n* = 29)	83.26 ± 9.16	58.41 ± 12.18 (*n* = 95)	*F* = 45.57, *p* < 0.001^2^ (ab, ac, ad, ae, bd, cd, de)
Clinical characteristics						
Number of lifetime depressive episodes, M ± s.d.	6.23 ± 4.3	8.64 ± 8.57 (*n* = 50)	7.96 ± 9.53 (*n* = 26)	2.55 ± 3.51	4.53 ± 4.83 (*n* = 89)	*F* = 7.01, *p* < 0.001^2^ (ad, bd, be, cd, ce, de)
Lifetime duration of depressive episodes (months), M ± s.d.	33.85 ± 46.26 (*n* = 34)	60.05 ± 92.86 (*n* = 42)	51.27 ± 66.96 (*n* = 26)	38.46 ± 58.41 (*n* = 34)	58.77 ± 94.62 (*n* = 74)	*F* = 0.915, *p* = 0.456^2^
Number of lifetime (hypo-)manic episodes, M ± s.d.	4.21 ± 3.11	6.22 ± 8.37 (*n* = 50)	5.81 ± 6.57 (*n* = 27)	n/a	n/a	*F* = 1.08, *p* = 0.344^2^
Lifetime duration of (hypo-)manic episodes (months), M ± s.d.	10.23 ± 12.26 (*n* = 34)	15.33 ± 34.34 (*n* = 42)	30.45 ± 54.07 (*n* = 25)	n/a	n/a	*F* = 2.46, *p* = 0.091^2^
Number of lifetime hospitalizations, M ± s.d.	3.53 ± 3.25 (*n* = 38)	4.12 ± 3.51 (*n* = 52)	3.50 ± 2.72 (*n* = 28)	0.84 ± 1.13	2.15 ± 2.36 (*n* = 96)	*F* = 10.62, *p* < 0.001^2^ (ad, ae, bd, be, cd, ce, de)
Lifetime duration of hospitalization (weeks), M ± s.d.	34.13 ± 36.83 (*n* = 38)	35.34 ± 34.02 (*n* = 50)	29.26 ± 32.94 (*n* = 28)	7.14 ± 10.8	14.43 ± 16.29 (*n* = 95)	*F* = 10.98, *p* < 0.001^2^ (ad, ae, bd, be, cd, ce, de)
Age of onset (years), M ± s.d.	22.51 ± 9.39	24.53 ± 11.7	25.59 ± 12.06 (*n* = 29)	29.89 ± 12.82	28.85 ± 12.78 (*n* = 97)	*F* = 3.12, *p* *=* 0.016^2^ (ad, ae, cd, ce)
Comorbid diagnoses (yes/no)	14/25	21/32	9/21	9/29	47/51	χ^2^ = 8.25, *p* = 0.083^3^
Current inpatient treatment (yes/no)	2/37	20/31	12/16	0/38	50/48	χ^2^ = 48.88, *p* < 0.001^3^ (ab, ac, ae, bd, cd, de)
Medication load Index, M ± s.d.	2.41 ± 1.68	2.83 ± 2.28	2.27 ± 1.86	0.58 ± 1.08	2.07 ± 1.71	*F* = 9.49, *p* < 0.001^2^ (ad, bd, be, cd, de)
Current lithium intake (yes/no)	13/26	13/40	8/22	1/37	4/94	χ^2^ = 30.78, *p* < 0.001^3^ (ad, ae, bd, be, cd, ce)
BD subtype (BD I/BD II)	21/18	28/25	19/11	n/a	n/a	χ^2^ = 0.940, *p* = 0.625^3^

BD, bipolar disorder; GAF, Global Assessment of Functioning; HC, healthy controls; HDRS, Hamilton Depression Rating Scale; MDD, major depressive disorder; s.d., standard deviation; YMRS, Young Mania Rating Scale; n, number; n/a, not applicable.

*Note:* All patients being allocated to a depressed group (BD or MDD) fulfilled the criteria of an acute or partially remitted episode, the same holds for the manic patients who fulfilled the criteria for a (hypo-)manic episode, according to a SCID-Interview. Due to missing information not all BD patients could be assigned to a subgroup. ^1^Significant differences in post-hoc *t* tests: ab = BD euthymic *v.* BD depressed, ac = BD euthymic *v.* BD (hypo-)manic, ad = BD euthymic *v.* MDD euthymic, ae = BD euthymic *v.* MDD depressed, bc = BD depressed *v.* BD (hypo-)manic, bd = BD depressed *v.* MDD euthymic, be = BD depressed *v.* MDD depressed, cd = BD (hypo-)manic *v.* MDD euthymic, ce = BD (hypo-)manic *v.* MDD depressed, de = MDD euthymic *v.* MDD depressed. ^2^One-way analysis of variance (ANOVA) *F*-test. ^3^Pearson χ^2^ test.

### DTI data acquisition

The DTI protocol and quality assurance protocol have already been described in detail elsewhere (Meinert et al., 2019; Vogelbacher et al., 2018). Data were acquired using 3T whole body MRI scanners. Detailed acquisition parameters are provided in online Supplementary material 3. For quality assurance, DTIPrep (Oguz et al., 2014) was used. Individual images of a given participant were eliminated if affected by artifacts. The included participants had 64.20 images on average (s.d. = 1.32, range: 56–65).

### Image processing

Preprocessing and analyses were implemented in FSL6.0.1 (http://fsl.fmrib.ox.ac.uk/fsl/fslwiki/) (Jenkinson, Beckmann, Behrens, Woolrich, & Smith, 2012; Smith et al., 2004; Woolrich et al., 2009). The DW images were corrected for motion and eddy current artifacts using ‘eddy’ from FSL (Andersson & Sotiropoulos, 2016), and *b*-vectors were rotated following eddy current correction. After automated skull stripping using the Brain Extraction Tool (BET) in FSL (Smith, 2002), the first *b_0_* image was used as reference for alignment. Diffusion tensor was estimated using ‘DTIFIT’ within FMRIB's Diffusion Toolbox (FDT) (Behrens et al., 2003) followed by the generation of tensor-derived maps. FA, mean diffusivity (MD), radial diffusivity (RD) and axial diffusivity (AD) were estimated for each voxel per participant (online Supplementary material 4).

### Analysis

IBM SPSS Statistics 27 (SPSS Inc., Chicago, IL, USA) was used for analyses of sociodemographic data. For DTI analyses, tract-based spatial statistics (TBSS) (Smith et al., 2006) were used to reduce registration misalignments and partial volume effects. Registration was performed using FMRIB's non-linear image registration tool and reslicing all FA images to the FMRIB58_FA template [1 × 1 × 1 mm^3^ Montreal Neurological Institute (MNI) standard space]. A WM skeleton was created using a threshold of 0.2 for the mean of all aligned FA images and overlaid onto each participant's registered FA image. By searching orthogonally from the skeleton for maximum FA values, we moved individual FA values onto the mean skeleton mask. To test for statistical significance, we used the nonparametric permutation testing implemented in ‘randomise’ from FSL (Winkler, Ridgway, Webster, Smith, & Nichols, 2014) with 5000 permutations. Threshold-Free Cluster Enhancement (TFCE) with default values provided by *–T_2_* option optimized for TBSS was used to correct for multiple comparisons. The 95th percentile of the null distribution of permutated input data of the maximum TFCE scores was used for determination of significance, correcting estimated cluster sizes for the family-wise error (FWE) at *p* < 0.05 (Smith & Nichols, 2009). For figures, mean FA of the significant clusters were extracted using ‘fslstats’ in FSL. The total intracranial volume (TIV) was extracted from T1 images using the Computational Anatomy Toolbox (CAT12, http://www.neuro.uni-jena.de/cat, v1720). Results focus on FA, as most studies report findings on this DTI measure. However, as the consideration of other DTI metrics can support the interpretation of the results, the same registration steps and analyses were performed on MD, RD, and AD as well. The results derived from these three metrics are summarized in online Supplementary material 5.

For correction of scanner differences between the two MRI scanners and due to a body-coil change at the Marburg site during data acquisition two dummy coded variables (Marburg pre body-coil change: yes/no; Marburg post body-coil change: yes/no) with Münster as reference category were calculated (Vogelbacher et al., 2018). These variables as well as age, sex, and TIV were included as nuisance variables in all analyses. Overall three analyses were conducted.

1. In a first step, to investigate diagnosis-specific differences in directional diffusion (hypotheses 1 and 2), a one-factorial Analysis of Covariance (ANCOVA) with FA as dependent variable and diagnosis (BD *v.* MDD *v.* HC) as independent variable was conducted. In case of significant effects, pairwise post hoc *t*-contrasts were performed between the three groups (analysis 1).

The following analyses, from which HC were excluded, aimed to further examine the differences in FA between BD and MDD patients in relation to their current mood (hypothesis 3).

1. To examine whether differences between BD and MDD patients were dependent on current symptom severity, the effect of diagnosis (BD *v.* MDD) on FA was reanalyzed together with the effect of depressive (HDRS) or manic (YMRS) symptom severity in two separate two-factorial ANCOVA models (analyses 2a, 2b). Due to missing values in the mentioned scales, three participants (*n* = 1 BD, *n* = 2 MDD) had to be excluded.
2. The third analysis aimed to explore the effect of mood state on the differences between BD and MDD patients in a categorical manner. Since there are naturally no manic patients within the MDD group, a subsample was created including only currently euthymic or depressed patients of both groups (BD: *n* = 39 euthymic, *n* = 53 depressed, MDD: *n* = 38 euthymic, *n* = 98 depressed). A 2 × 2 ANCOVA with FA as dependent variable and diagnosis (BD *v.* MDD) and mood state (euthymic *v.* depressed) as independent variables was conducted (analysis 3).

All analyses on patient groups were repeated adding the MedIndex, the number of lifetime depressive episodes as well as hospitalizations as nuisance variables in the model.

## Results

### Analysis 1: HC *v.* MDD *v.* BD

**Fig. 1.**
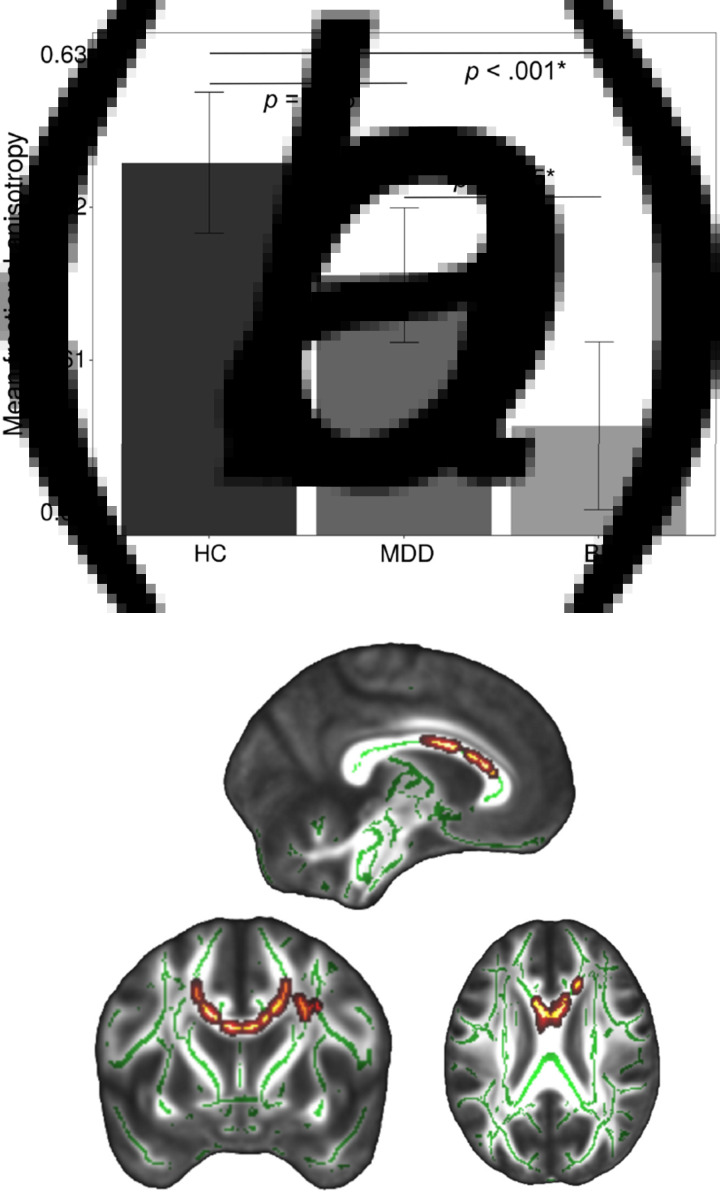
Main effect of diagnosis across bipolar disorder (BD), major depressive disorder (MDD), and healthy controls (HC). (*a*) Mean fractional anisotropy (FA) across HC, patients with MDD and patients with BD. The mean FA value was obtained from FA values of all the voxels that showed a significant main effect of diagnosis (*p_tfce-FWE_* < 0.05). Error bars represent 95% confidence intervals. *p* values were obtained from pairwise post hoc *t*-contrasts, asterisks indicate significant differences between groups. (*b*) Effect displayed on the FMRIB58 template. Highlighted areas represent voxels (using FSL's ‘fill’ command for better visualization), where a significant main effect of diagnosis on FA was detected (*p_tfce-FWE_* < 0.05). MNI coordinates for the section plane: *x* = −4, *y* = 5, *z* = 24.

**Fig. 2.**
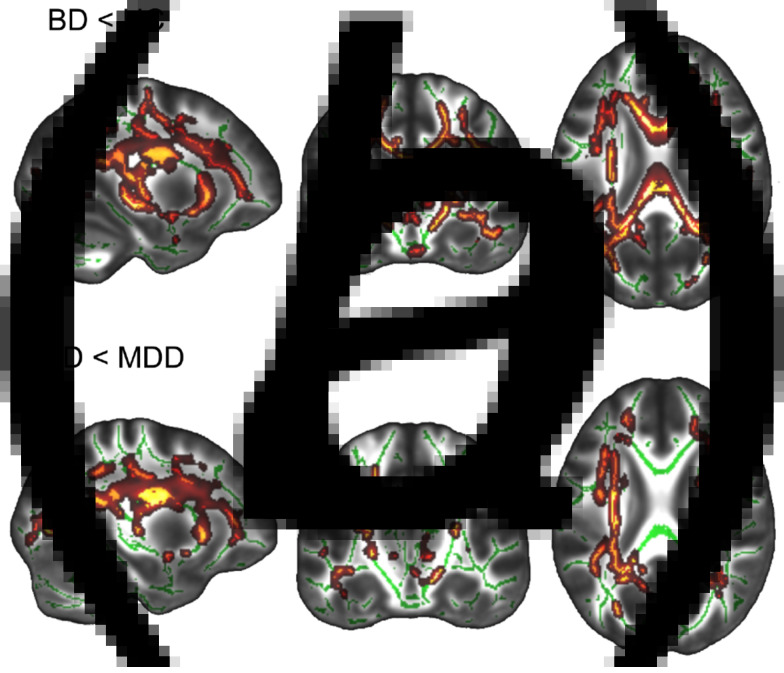
Reduced fractional anisotropy (FA) in patients with bipolar disorder (BD) compared to healthy controls (HC) and major depressive disorder (MDD). MNI coordinates for the section plane: *x* = −28, *y* = −17, *z* = 23. Highlighted areas represent voxels (using FSL's ‘fill’ command for better visualization), where significant differences between groups (*p_tfce-FWE_* < 0.05) were detected. (*a*) Reduced FA in patients with BD compared to HC. (*b*) Reduced FA in patients with BD compared to patients with MDD.

A significant main effect of diagnosis across BD, MDD, and HC on FA (*p_tfce-FWE_* = 0.003, total *k* = 2448 voxels in 13 clusters, peak voxel of largest cluster: *x* = −4, *y* = 5, *z* = 24, see online Supplementary Table S3 for the location and size of all significant clusters) was found (Fig. 1). Post hoc *t* tests revealed significantly reduced FA in the BD group compared to MDD and HC groups. In contrast, differences between MDD and HC groups only reached a trend level of significance (*p_tfce-FWE_* = 0.095). Specifically, BD patients had significantly lower FA values compared to HC in one large bilateral cluster (*p_tfce-FWE_* < 0.001, *k* = 38 575 voxels, peak voxel: *x* = −14, *y* = 11, *z* = 28) comprising the forceps minor and major, the inferior fronto-occipital fasciculi, the inferior longitudinal fasciculi, and bilateral superior longitudinal fasciculi among other regions (Fig. 2*a*, online Supplementary Table S4). The effect was most probably located in the forceps minor of the corpus callosum. Compared to MDD patients, BD patients showed reduced FA (*p_tfce-FWE_* = 0.005, total *k* = 17 689 voxels in eight clusters, peak voxel of largest cluster: *x* = −28, *y* = −17, *z* = 23) in several WM tracts including, amongst others, bilateral anterior thalamic radiation, left inferior fronto-occipital fasciculus, left inferior longitudinal fasciculus and left superior longitudinal fasciculus including the temporal part (Fig. 2*b*). There were no significant increases in FA in BD as compared to MDD or HC (all *p_tfce_* > 0.978). The differences between MDD and BD remained significant even after correcting for the MedIndex and the number of depressive episodes and hospitalizations, on top of age, sex, TIV, and scanner differences (online Supplementary Tables S3 and S4). For RD, we found significantly increased values in BD compared to MDD and HC, but again no significant differences between MDD and HC. No effects were found for MD and AD (online Supplementary material 5).

### Analysis 2a: ANCOVA with diagnosis, HDRS scores, and their interaction

In the model with diagnosis and HDRS scores as independent variables, only the main effect of diagnosis proved significant (*p_tfce-FWE_* = 0.004). As before, post hoc *t* tests showed a reduction of FA in BD compared to MDD (*p_tfce-FWE_* = 0.006, total *k* = 20 562 voxels in five clusters, peak voxel of largest cluster: *x* = −24, *y* = −24, *z* = 35) (online Supplementary Fig. S1*a*), affecting the same tracts as in analysis 1 (online Supplementary Table S4). There was neither a significant main effect of HDRS scores (*p_tfce-FWE_* = 0.388), nor a significant HDRS × diagnosis interaction (*p_tfce-FWE_* = 0.781).

### Analysis 2b: ANCOVA with diagnosis, YMRS scores, and their interaction

The model with diagnosis and YMRS scores as independent variables again revealed a significant main effect of diagnosis (*p_tfce-FWE_* = 0.014), which was further determined by post hoc *t*-contrasts as a reduction in FA in BD patients (*p_tfce-FWE_* = 0.008, total *k* = 16 129 voxels in three clusters, peak voxel of largest cluster: *x* = −24, *y* = −25, *z* = 33) (online Supplementary Fig. S1*b*). The effect was located in several WM tracts, consistent with the results of previous analyses (online Supplementary Table S4). Neither the main effect of YMRS scores (*p_tfce-FWE_* = 0.931) nor the YMRS × diagnosis interaction was significant (*p_tfce-FWE_* = 0.959).

Main results of analyses 2a and 2b remained unchanged after inclusion of clinical covariates (MedIndex and the number of depressive episodes and hospitalizations) in the model (online Supplementary Tables S3 and S4). For RD, we also found a significant main effect of diagnosis in both models, but no significant main effect of HDRS or YMRS scores or an interaction (online Supplementary material 5).

### Analysis 3: ANCOVA with diagnosis, mood state, and their interaction in the subsample without manic BD patients

In the subsample including only patients in a current euthymic or depressed mood state, neither an effect of mood state (*p_tfce-FWE_* = 0.906) nor an interaction of diagnosis and mood state (*p_tfce-FWE_* = 0.705) on FA reached level of significance. However, a significant main effect of diagnosis emerged (*p_tfce-FWE_* = 0.039). Post hoc *t*-contrasts again confirmed the previously observed reduction of FA in BD compared to MDD (*p_tfce-FWE_* = 0.036, total *k* = 2636 voxels in seven clusters, peak voxel of largest cluster: *x* = −19, *y* = −33, *z* = 32). Effects were primarily located in the left corticospinal tract and the left cingulate gyrus (online Supplementary Fig. S2). When MedIndex and the number of depressive episodes and hospitalizations were added to the model, the main effect of diagnosis remained significant (online Supplementary Tables S3 and S4). No significant effects were found for AD, MD, and RD (online Supplementary material 5). An additional analysis revealed no significant differences in any DTI measures between manic, depressed, and euthymic BD patients (see online Supplementary material 6).

## Discussion

The present study found widespread alterations in WM integrity in BD patients compared to MDD patients and HC, confirming our hypothesis. MDD patients, on the other hand, had lower FA values than HC, which, however, only reached a trend level of significance. All findings remained unchanged after controlling for medication load and the number of depressive episodes and hospitalizations, supporting the conclusion of a robust change in WM microstructure in BD compared to both HC and MDD. Exploratory investigation regarding the role of patients' current mood state revealed that it did not affect the differences between patient groups. This was reflected in non-significant interactions between diagnosis and mood, both in a dimensional assessment of mood via depressive (HDRS) and manic (YMRS) symptom severity and in a categorical examination of euthymic and depressed state after excluding manic BD patients.

The present changes in WM microstructure in BD were widespread, with reduced FA and increased RD in multiple WM tracts not restricted to fronto-temporal regions. Our findings therefore point toward extensive and global rather than localized WM changes in BD, compared to both HC and MDD. Therefore, the present results might be interpreted in terms of global structural disconnectivity in BD, as has been discussed for schizophrenia (Kelly et al., 2018). This conclusion is also supported by other studies comparing BD with HC, which reported affected tracts in frontal and temporal regions classically associated with emotion processing and regulation, but also other major WM pathways beyond these (Jenkins et al., 2016; Nortje, Stein, Radua, Mataix-Cols, & Horn, 2013; Wise et al., 2016). Among the former, impaired integrity of the cingulum is particularly associated with the pathophysiology of BD. As the most prominent pathway in the limbic system, it is implicated in numerous processes whose impairment is characteristic of BD, such as emotion processing and regulation as well as reward processing, cognition, and attention (Bracht, Linden, & Keedwell, 2015; Duarte et al., 2016; Mertse et al., 2022). In MDD, impaired microstructure in the cingulum has also already been associated with related specific symptoms such as anhedonia (Keedwell et al., 2012; Yang et al., 2017) or rumination (Zhu et al., 2012). Similarly, disrupted microstructure in other major WM pathways has been related to specific aberrant functions and behaviors in BD, such as the CST with psychomotor retardation and agitation (Bracht et al., 2018; Ji et al., 2017; Sacchet et al., 2014) or the superior and inferior longitudinal fascicles with impairments in language, cognition, or visuospatial functions (El Nagar et al., 2021; Ji et al., 2017; Magioncalda et al., 2016; Poletti et al., 2015; Sprooten et al., 2016). Overall, however, no definite interpretation of the reduced FA in these pathways can yet be made. Therefore, we interpret the differences as an indication of greater overall impairment in BD compared with MDD. Contrary to the widespread FA reductions in BD, our results indicate no large – albeit at a trend level of significance – differences between MDD and HC, which contradicts our hypothesis. This finding is in line with previous studies arguing that the inconsistently found differences between MDD patients and HC might be influenced by clinical heterogeneity or other clinical factors rather than the diagnosis itself (Choi et al., 2014; Meinert et al., 2019; Olvet et al., 2016). However, the smaller differences in MDD patients compared to HC support the notion that the neuropathological characteristics of MDD may differ from those of BD (De Almeida & Phillips, 2013; Koshiyama et al., 2020; Sexton, Mackay, & Ebmeier, 2009). In addition to the present brain structural differences between BD and MDD, this assumption is also supported by differences in clinical phenotype between the two groups, showing a more severe disease course for BD (Table 1).

So far, only one study exists that is comparable to our work, examining differences in FA between euthymic and depressed BD and MDD patients (Matsuoka et al., 2017). However, the authors did not include currently manic patients in their sample. Moreover, they did not examine the effect of depressive symptom severity in more detail but only included depressive symptom severity as a covariate in the model. Thus, ours was the first study to combine a categorical and a dimensional approach to examine the influence of current mood on the difference between BD and MDD regarding WM microstructure, including patients in the euthymic, depressed, and manic mood state.

Using the categorical approach, it was not possible to examine the effect of all mood states on the difference between BD and MDD in one model because there was no group of manic MDD patients. However, there were no differences within the BD group when the three mood states were tested against each other (online Supplementary material 6). Accordingly, the differences between BD and MDD emerged independently of the inclusion or exclusion of manic BD patients and unaffected by current euthymic or depressed mood state of either disorder. Complementing this analysis, dimensional measures of current depressive and manic symptoms allowed us to capture the full spectrum of symptoms, including subclinical manic symptoms in MDD (see online Supplementary Fig. S1*b*) or subclinical depressive symptoms in euthymic states. Central to this approach was that our sample included highly symptomatic patients as well as patients in partial remission and euthymic states, allowing us to analyze an adequate variance in symptom severity.

Although we cannot rule out that the lack of interaction between diagnosis and mood state could result from the rather small sample size per group, overall, our results suggest that reduced FA in BD *v*. MDD, previously observed in depressed patients (Deng et al., 2018; Lan et al., 2020; Repple et al., 2017; Vai et al., 2020; Versace et al., 2010), may be generalized to the entire symptom spectrum of BD. The disrupted WM microstructure does not appear to be caused by state-dependent changes, but instead seems to be a rather stable neurobiological alteration that distinguishes the disorder from MDD, regardless of depressive or manic symptoms. Considering the high heritability of both BD (McGuffin et al., 2003) and WM microstructure (Chiang et al., 2009; Kochunov et al., 2010), these state-independent differences suggest the involvement of genetic factors. This assumption is also supported by studies that found reductions in FA even in unaffected subjects at familial risk of BD and interpreted these in terms of a potential endophenotype of BD (Foley et al., 2018; Hu, Stavish, Leibenluft, & Linke, 2020; Sarlçiçek et al., 2016; Sprooten et al., 2011). Thus, DTI metrics may have the potential for being used as a diagnostic tool for BD in the future, possibly as part of a multimodality diagnostic imaging approach including machine learning algorithms (Bürger et al., 2017; Grotegerd et al., 2013; Lan et al., 2020; Vai et al., 2020; Versace et al., 2010).

The neurobiological interpretation of these observed reductions in FA is not yet understood in detail and remains challenging. FA represents the strength of anisotropy and is considered as a measure of WM integrity. However, various factors are discussed in relation to FA reductions, including demyelination, membrane permeability, axonal count and diameter, and crossing of fibers (Alexander, Lee, Lazar, & Field, 2007; Feldman, Yeatman, Lee, Barde, & Gaman-Bean, 2010; Jones et al., 2013). When drawing conclusions about the nature of microstructural changes, additional consideration should also be given to the other DTI metrics, for which we observed an increase in RD, whereas AD and MD showed no differences.

A few limitations of this study should be noted. First, the results of this study are cross-sectional in nature, making it impossible to draw causal conclusions. Inferences about whether the observed effects are state-dependent or persistent differences should be drawn with caution. Comprehensively answering this question requires future longitudinal studies examining the same patients in varying mood states (Benedetti et al., 2011b; Phillips, 2019). Second, the majority of patients were taking psychotropic medications, with differences between BD and MDD. Although all effects remained unchanged after accounting for current medication load, we cannot completely exclude the possibility that – especially past – medication use confounded the effects. However, in support of our findings, a normalizing effect has already been reported for lithium, counteracting and possibly even concealing changes in WM microstructure in BD (Favre et al., 2019; Hafeman, Chang, Garrett, Sanders, & Phillips, 2012). This supports our results as we found significant effects even though some of the BD patients received lithium medication. Finally, although we controlled for the number of depressive episodes and hospitalizations, there are many more variables associated with disease course that may have an effect on microstructural WM changes (Favre et al., 2019; Koshiyama et al., 2020; Repple et al., 2017; Yang et al., 2019).

Taken together, our results contribute to a deeper understanding of WM microstructure impairment in BD. To the best of our knowledge, no previous study has investigated differences between BD and MDD with detailed consideration of patients' current mood. Although interpretation should be made with caution due to the cross-sectional design of the study, our results support the existence of global microstructural WM disruptions in BD patients as compared to MDD patients, unaffected by current affective state and symptom severity. To further investigate the transient *v*. persistent nature of WM integrity impairments in BD, future studies using longitudinal designs are needed.

## Supplementary material

For supplementary material accompanying this paper visit https://doi.org/10.1017/S0033291722001490. click here to view supplementary material

## References

[ref1] Alexander, A. L., Lee, J. E., Lazar, M., & Field, A. S. (2007). Diffusion tensor imaging of the brain. Neurotherapeutics, 4(3), 316–329. 10.1016/j.nurt.2007.05.011. 17599699 PMC2041910

[ref2] Andersson, J. L. R., & Sotiropoulos, S. N. (2016). An integrated approach to correction for off-resonance effects and subject movement in diffusion MR imaging. NeuroImage, 125, 1063–1078. 10.1016/j.neuroimage.2015.10.019. 26481672 PMC4692656

[ref3] Behrens, T. E. J., Woolrich, M. W., Jenkinson, M., Johansen-Berg, H., Nunes, R. G., Clare, S., … Smith, S. M. (2003). Characterization and propagation of uncertainty in diffusion-weighted MR imaging. Magnetic Resonance in Medicine, 50(5), 1077–1088. 10.1002/mrm.10609. 14587019

[ref4] Benedetti, F., Absinta, M., Rocca, M. A., Radaelli, D., Poletti, S., Bernasconi, A., … Filippi, M. (2011a). Tract-specific white matter structural disruption in patients with bipolar disorder. Bipolar Disorders, 13(4), 414–424. 10.1111/j.1399-5618.2011.00938.x. 21843281

[ref5] Benedetti, F., Yeh, P. H., Bellani, M., Radaelli, D., Nicoletti, M. A., Poletti, S., … Brambilla, P. (2011b). Disruption of white matter integrity in bipolar depression as a possible structural marker of illness. Biological Psychiatry, 69(4), 309–317. 10.1016/j.biopsych.2010.07.028. 20926068

[ref6] Berk, M., Dodd, S., Callaly, P., Berk, L., Fitzgerald, P., de Castella, A. R., … Kulkarni, J. (2007). History of illness prior to a diagnosis of bipolar disorder or schizoaffective disorder. Journal of Affective Disorders, 103(1–3), 181–186. 10.1016/j.jad.2007.01.027. 17324469

[ref7] Bracht, T., Linden, D., & Keedwell, P. (2015). A review of white matter microstructure alterations of pathways of the reward circuit in depression. Journal of Affective Disorders, 187, 45–53. 10.1016/J.JAD.2015.06.041. 26318270

[ref8] Bracht, T., Steinau, S., Federspiel, A., Schneider, C., Wiest, R., & Walther, S. (2018). Physical activity is associated with left corticospinal tract microstructure in bipolar depression. NeuroImage: Clinical, 20, 939–945. 10.1016/J.NICL.2018.09.033. 30308380 PMC6178191

[ref9] Bürger, C., Redlich, R., Grotegerd, D., Meinert, S., Dohm, K., Schneider, I., … Dannlowski, U. (2017). Differential abnormal pattern of anterior cingulate gyrus activation in unipolar and bipolar depression: An fMRI and pattern classification approach. Neuropsychopharmacology, 42, 1399–1408. 10.1038/npp.2017.36. 28205606 PMC5436122

[ref10] Chen, G., Hu, X., Li, L., Huang, X., Lui, S., Kuang, W., … Gong, Q. (2016). Disorganization of white matter architecture in major depressive disorder: A meta-analysis of diffusion tensor imaging with tract-based spatial statistics. Scientific Reports, 6, 21825. 10.1038/srep21825. 26906716 PMC4764827

[ref11] Chiang, M.-C., Barysheva, M., Shattuck, D. W., Lee, A. D., Madsen, S. K., Avedissian, C., … Thompson, P. M. (2009). Genetics of brain fiber architecture and intellectual performance. The Journal of Neuroscience, 29(7), 2212. 10.1523/JNEUROSCI.4184-08.2009. 19228974 PMC2773128

[ref12] Choi, K. S., Holtzheimer, P. E., Franco, A. R., Kelley, M. E., Dunlop, B. W., Hu, X. P., & Mayberg, H. S. (2014). Reconciling variable findings of white matter integrity in major depressive disorder. Neuropsychopharmacology, 39(6), 1332–1339. 10.1038/npp.2013.345. 24352368 PMC3988550

[ref13] Cui, Y., Dong, J., Yang, Y., Yu, H., Li, W., Liu, Y., … Jiang, T. (2020). White matter microstructural differences across major depressive disorder, bipolar disorder and schizophrenia: A tract-based spatial statistics study. Journal of Affective Disorders, 260, 281–286. 10.1016/j.jad.2019.09.029. 31521864

[ref14] De Almeida, J. R. C., & Phillips, M. L. (2013). Distinguishing between unipolar depression and bipolar depression: Current and future clinical and neuroimaging perspectives. Biological Psychiatry, 73(2), 111–118. 10.1016/j.biopsych.2012.06.010. 22784485 PMC3494754

[ref15] Deng, F., Wang, Y., Huang, H., Niu, M., Zhong, S., Zhao, L., … Huang, R. (2018). Abnormal segments of right uncinate fasciculus and left anterior thalamic radiation in major and bipolar depression. Progress in Neuro-Psychopharmacology and Biological Psychiatry, 81, 340–349. 10.1016/j.pnpbp.2017.09.006. 28912043

[ref16] Duarte, J. A., De Araújo e Silva, J. Q., Goldani, A. A., Massuda, R., & Gama, C. S. (2016). Neurobiological underpinnings of bipolar disorder focusing on findings of diffusion tensor imaging: A systematic review. Revista Brasileira de Psiquiatria, 38(2), 167–175. 10.1590/1516-4446-2015-1793. 27007148 PMC7111360

[ref17] Dvorak, J., Hilke, M., Trettin, M., Wenzler, S., Hagen, M., Ghirmai, N., … Oertel, V. (2019). Aberrant brain network topology in fronto-limbic circuitry differentiates euthymic bipolar disorder from recurrent major depressive disorder. Brain and Behavior, 9(6), e01257. 10.1002/brb3.1257. 31066228 PMC6576154

[ref18] El Nagar, Z., El Shahawi, H. H., Effat, S. M., El Sheikh, M. M., Adel, A., Ibrahim, Y. A., … Aufa, O. M. (2021). Single episode brief psychotic disorder versus bipolar disorder: A diffusion tensor imaging and executive functions study. Schizophrenia Research Cognition, 27, 100214. 10.1016/J.SCOG.2021.100214. 34557386 PMC8446778

[ref19] Favre, P., Pauling, M., Stout, J., Hozer, F., Sarrazin, S., Abé, C., … Houenou, J. (2019). Widespread white matter microstructural abnormalities in bipolar disorder: Evidence from mega- and meta-analyses across 3033 individuals. Neuropsychopharmacology, 44(13), 2285–2293. 10.1038/s41386-019-0485-6. 31434102 PMC6898371

[ref20] Feldman, H. M., Yeatman, J. D., Lee, E. S., Barde, L. H. F., & Gaman-Bean, S. (2010). Diffusion tensor imaging: A review for pediatric researchers and clinicians. Journal of Developmental and Behavioral Pediatrics, 31(4), 346–356. 10.1097/DBP.0b013e3181dcaa8b. 20453582 PMC4245082

[ref21] Foley, S., Bracher-Smith, M., Tansey, K., Harrison, J., Parker, G., & Caseras, X. (2018). Fractional anisotropy of the uncinate fasciculus and cingulum in bipolar disorder type I, type II, unaffected siblings and healthy controls. The British Journal of Psychiatry : The Journal of Mental Science, 213(3), 548–554. 10.1192/BJP.2018.101. 30113288 PMC6130806

[ref22] Grande, I., Berk, M., Birmaher, B., & Vieta, E. (2016). Bipolar disorder. The Lancet, 387(10027), 1561–1572. 10.1016/S0140-6736(15)00241-X. 26388529

[ref23] Grotegerd, D., Suslow, T., Bauer, J., Ohrmann, P., Arolt, V., Stuhrmann, A., … Dannlowski, U. (2013). Discriminating unipolar and bipolar depression by means of fMRI and pattern classification: A pilot study. European Archives of Psychiatry and Clinical Neuroscience, 263(2), 119–131. 10.1007/s00406-012-0329-4. 22639242

[ref24] Hafeman, D. M., Chang, K. D., Garrett, A. S., Sanders, E. M., & Phillips, M. L. (2012). Effects of medication on neuroimaging findings in bipolar disorder: An updated review. Bipolar Disorders, 14(4), 375–410. 10.1111/j.1399-5618.2012.01023.x. 22631621

[ref25] Hamilton, M. (1960). A rating scale for depression. Journal of Neurology, Neurosurgery, and Psychiatry, 23, 56–62. 14399272 10.1136/jnnp.23.1.56 PMC495331

[ref26] Han, K. M., De Berardis, D., Fornaro, M., & Kim, Y. K. (2019). Differentiating between bipolar and unipolar depression in functional and structural MRI studies. Progress in Neuro-Psychopharmacology and Biological Psychiatry, 91, 20–27. 10.1016/j.pnpbp.2018.03.022. 29601896

[ref27] Hassel, S., Almeida, J. R. C., Kerr, N., Nau, S., Ladouceur, C. D., Fissell, K., … Phillips, M. L. (2008). Elevated striatal and decreased dorsolateral prefrontal cortical activity in response to emotional stimuli in euthymic bipolar disorder: No associations with psychotropic medication load. Bipolar Disorders, 10(8), 916–927. 10.1111/j.1399-5618.2008.00641.x. 19594507 PMC2711546

[ref28] Hirschfeld, R. M. A., Lewis, L., & Vornik, L. A. (2003). Perceptions and impact of bipolar disorder: How far have we really come? Results of the National Depressive and Manic-Depressive Association 2000 Survey of individuals with bipolar disorder. Journal of Clinical Psychiatry, 64(2), 161–174. 10.4088/JCP.V64N0209. 12633125

[ref29] Ho, D. E., Imai, K., King, G., & Stuart, E. A. (2011). MatchIt: Nonparametric preprocessing for parametric causal inference. Journal of Statistical Software, 42(8), 1–28. 10.18637/jss.v042.i08.

[ref30] Hu, R., Stavish, C., Leibenluft, E., & Linke, J. O. (2020). White matter microstructure in individuals with and at risk for bipolar disorder: Evidence for an endophenotype from a voxel-based meta-analysis. Biological Psychiatry: Cognitive Neuroscience and Neuroimaging, 5(12), 1104–1113. 10.1016/j.bpsc.2020.06.007. 32839153 PMC11102922

[ref31] Jenkins, L. M., Barba, A., Campbell, M., Lamar, M., Shankman, S. A., Leow, A. D., … Langenecker, S. A. (2016). Shared white matter alterations across emotional disorders: A voxel-based meta-analysis of fractional anisotropy. NeuroImage: Clinical, 12, 1022–1034. 10.1016/j.nicl.2016.09.001. 27995068 PMC5153602

[ref32] Jenkinson, M., Beckmann, C. F., Behrens, T. E. J., Woolrich, M. W., & Smith, S. M. (2012). FSL. NeuroImage, 62(2), 782–790. 10.1016/j.neuroimage.2011.09.015. 21979382

[ref33] Ji, A., Godwin, D., Rutlin, J., Kandala, S., Shimony, J. S., & Mamah, D. (2017). Tract-based analysis of white matter integrity in psychotic and nonpsychotic bipolar disorder. Journal of Affective Disorders, 209, 124–134. 10.1016/J.JAD.2016.11.038. 27914246

[ref34] Jones, D. K., Knösche, T. R., & Turner, R. (2013). White matter integrity, fiber count, and other fallacies: The do's and don'ts of diffusion MRI. NeuroImage, 73, 239–254. 10.1016/j.neuroimage.2012.06.081. 22846632

[ref35] Keedwell, P. A., Chapman, R., Christiansen, K., Richardson, H., Evans, J., & Jones, D. K. (2012). Cingulum white matter in young women at risk of depression: The effect of family history and anhedonia. BPS, 72, 296–302. 10.1016/j.biopsych.2012.01.022. 22386005

[ref36] Kelly, S., Jahanshad, N., Zalesky, A., Kochunov, P., Agartz, I., Alloza, C., … Donohoe, G. (2018). Widespread white matter microstructural differences in schizophrenia across 4322 individuals: Results from the ENIGMA Schizophrenia DTI Working Group. Molecular Psychiatry, 23(5), 1261–1269. 10.1038/mp.2017.170. 29038599 PMC5984078

[ref37] Kircher, T., Wöhr, M., Nenadic, I., Schwarting, R., Schratt, G., Alferink, J., … Dannlowski, U. (2019). Neurobiology of the major psychoses: A translational perspective on brain structure and function – the FOR2107 consortium. European Archives of Psychiatry and Clinical Neuroscience, 269(8), 949–962. 10.1007/s00406-018-0943-x. 30267149

[ref38] Kochunov, P., Glahn, D. C., Lancaster, J. L., Winkler, A. M., Smith, S., Thompson, P. M., … Blangero, J. (2010). Genetics of microstructure of cerebral white matter using diffusion tensor imaging. NeuroImage, 53(3), 1109–1116. 10.1016/J.NEUROIMAGE.2010.01.078. 20117221 PMC2888778

[ref39] Koshiyama, D., Fukunaga, M., Okada, N., Morita, K., Nemoto, K., Usui, K., … Hashimoto, R. (2020). White matter microstructural alterations across four major psychiatric disorders: Mega-analysis study in 2937 individuals. Molecular Psychiatry, 25(4), 883–895. 10.1038/s41380-019-0553-7. 31780770 PMC7156346

[ref40] Lan, M. J., Rubin-Falcone, H., Sublette, M. E., Oquendo, M. A., Stewart, J. W., Hellerstein, D. J., … Mann, J. J. (2020). Deficits of white matter axial diffusivity in bipolar disorder relative to major depressive disorder: No relationship to cerebral perfusion or body mass index. Bipolar Disorders, 22(3), 296–302. 10.1111/bdi.12845. 31604361

[ref41] Magioncalda, P., Martino, M., Conio, B., Piaggio, N., Teodorescu, R., Escelsior, A., … Amore, M. (2016). Patterns of microstructural white matter abnormalities and their impact on cognitive dysfunction in the various phases of type I bipolar disorder. Journal of Affective Disorders, 193, 39–50. 10.1016/j.jad.2015.12.050. 26766032

[ref42] Manelis, A., Soehner, A., Halchenko, Y. O., Satz, S., Ragozzino, R., Lucero, M., … Versace, A. (2021). White matter abnormalities in adults with bipolar disorder type-II and unipolar depression. Scientific Reports, 11, 7541. 10.1038/s41598-021-87069-2. 33824408 PMC8024340

[ref43] Masuda, Y., Okada, G., Takamura, M., Shibasaki, C., Yoshino, A., Yokoyama, S., … Okamoto, Y. (2020). White matter abnormalities and cognitive function in euthymic patients with bipolar disorder and major depressive disorder. Brain and Behavior, 10(12), e01868. 10.1002/brb3.1868. 33009714 PMC7749556

[ref44] Matsuoka, K., Yasuno, F., Kishimoto, T., Yamamoto, A., Kiuchi, K., Kosaka, J., … Kudo, T. (2017). Microstructural differences in the corpus callosum in patients with bipolar disorder and major depressive disorder. Journal of Clinical Psychiatry, 78(1), 99–104. 10.4088/JCP.15m09851. 27574839

[ref45] McGuffin, P., Rijsdijk, F., Andrew, M., Sham, P., Katz, R., & Cardno, A. (2003). The heritability of bipolar affective disorder and the genetic relationship to unipolar depression. Archives of General Psychiatry, 60(5), 497–502. 10.1001/archpsyc.60.5.497. 12742871

[ref46] Meinert, S., Repple, J., Nenadic, I., Krug, A., Jansen, A., Grotegerd, D., … Dannlowski, U. (2019). Reduced fractional anisotropy in depressed patients due to childhood maltreatment rather than diagnosis. Neuropsychopharmacology, 44(12), 2065–2072. 10.1038/s41386-019-0472-y. 31382267 PMC6897978

[ref47] Mertse, N., Denier, N., Walther, S., Breit, S., Grosskurth, E., Federspiel, A., … Bracht, T. (2022). Associations between anterior cingulate thickness, cingulum bundle microstructure, melancholia and depression severity in unipolar depression. Journal of Affective Disorders, 301, 437–444. 10.1016/J.JAD.2022.01.035. 35026360

[ref48] Metin, S., Altuglu, T., Metin, B., & Tarhan, K. (2020). Anatomical connectivity changes can differentiate patients with unipolar depression and bipolar disorders. Psychiatry and Behavioral Sciences, 10(2), 72. 10.5455/pbs.20200228015036.

[ref49] Nortje, G., Stein, D. J., Radua, J., Mataix-Cols, D., & Horn, N. (2013). Systematic review and voxel-based meta-analysis of diffusion tensor imaging studies in bipolar disorder. Journal of Affective Disorders, 150(2), 192–200. 10.1016/j.jad.2013.05.034. 23810479

[ref50] Oguz, I., Farzinfar, M., Matsui, J., Budin, F., Liu, Z., Gerig, G., … Styner, M. (2014). DTIPrep: Quality control of diffusion-weighted images. Frontiers in Neuroinformatics, 8, 4. 10.3389/fninf.2014.00004. 24523693 PMC3906573

[ref51] Olvet, D. M., Delaparte, L., Yeh, F. C., Delorenzo, C., McGrath, P. J., Weissman, M. M., … Parsey, R. V. (2016). A comprehensive examination of white matter tracts and connectometry in major depressive disorder. Depression and Anxiety, 33(1), 56–65. 10.1002/da.22445. 26477532 PMC4701622

[ref52] Phillips, M. L. (2019). Neural markers that distinguish bipolar disorder from major depressive disorder: Moving closer to a reality. Biological Psychiatry: Cognitive Neuroscience and Neuroimaging, 4(4), 328–330. 10.1016/j.bpsc.2019.01.010. 30961832 PMC8444227

[ref53] Phillips, M. L., & Kupfer, D. J. (2013). Bipolar disorder 2 – bipolar disorder diagnosis: Challenges and future directions. The Lancet, 381(9878), 1663–1671. 10.1016/S0140-6736(13)60989-7. PMC5858935 23663952

[ref54] Phillips, M. L., & Swartz, H. A. (2014). A critical appraisal of neuroimaging studies of bipolar disorder: Toward a new conceptualization of underlying neural circuitry and a road map for future research. American Journal of Psychiatry, 171(8), 829–843. 10.1176/appi.ajp.2014.13081008. 24626773 PMC4119497

[ref55] Poletti, S., Bollettini, I., Mazza, E., Locatelli, C., Radaelli, D., Vai, B., … Benedetti, F. (2015). Cognitive performances associate with measures of white matter integrity in bipolar disorder. Journal of Affective Disorders, 174, 342–352. 10.1016/j.jad.2014.12.030. 25553397

[ref56] Redlich, R., Almeida, J. R., Grotegerd, D., Opel, N., Kugel, H., Heindel, W., … Dannlowski, U. (2014). Brain morphometric biomarkers distinguishing unipolar and bipolar depression: A voxel-based morphometry-pattern classification approach. JAMA Psychiatry, 71(11), 1222–1230. 10.1001/jamapsychiatry.2014.1100. 25188810 PMC5538312

[ref57] Repple, J., Meinert, S., Grotegerd, D., Kugel, H., Redlich, R., Dohm, K., … Dannlowski, U. (2017). A voxel-based diffusion tensor imaging study in unipolar and bipolar depression. Bipolar Disorders, 19(1), 23–31. 10.1111/bdi.12465. 28239946

[ref58] Sacchet, M. D., Livermore, E. E., Iglesias, J. E., Glover, G. H., & Gotlib, I. H. (2015). Subcortical volumes differentiate major depressive disorder, bipolar disorder, and remitted major depressive disorder. Journal of Psychiatric Research, 68, 91–98. 10.1016/j.jpsychires.2015.06.002. 26228406 PMC11887997

[ref59] Sacchet, M. D., Prasad, G., Foland-Ross, L. C., Joshi, S. H., Hamilton, J. P., Thompson, P. M., … Gotlib, I. H. (2014). Structural abnormality of the corticospinal tract in major depressive disorder. Biology of Mood and Anxiety Disorders, 4(1), 8. 10.1186/2045-5380-4-8. 25295159 PMC4187017

[ref60] Sarlçiçek, A., Zorlu, N., Yalln, N., Hldlroǧlu, C., Çavuşoǧlu, B., Ceylan, D., … Özerdem, A. (2016). Abnormal white matter integrity as a structural endophenotype for bipolar disorder. Psychological Medicine, 46(7), 1547–1558. 10.1017/S0033291716000180. 26947335

[ref61] Saß, H., & Wittchen, H.-U. (2003). Diagnostische Kriterien des Diagnostischen und Statistischen Manuals Psychischer Störungen, DSM-IV-TR. Göttingen: Hogrefe.

[ref62] Sexton, C. E., Mackay, C. E., & Ebmeier, K. P. (2009). A systematic review of diffusion tensor imaging studies in affective disorders. Biological Psychiatry, 66(9), 814–823. 10.1016/j.biopsych.2009.05.024. 19615671

[ref63] Smith, S. M. (2002). Fast robust automated brain extraction. Human Brain Mapping, 17(3), 143–155. 10.1002/hbm.10062. 12391568 PMC6871816

[ref64] Smith, S. M., Jenkinson, M., Johansen-Berg, H., Rueckert, D., Nichols, T. E., Mackay, C. E., … Behrens, T. E. J. (2006). Tract-based spatial statistics: Voxelwise analysis of multi-subject diffusion data. NeuroImage, 31(4), 1487–1505. 10.1016/j.neuroimage.2006.02.024. 16624579

[ref65] Smith, S. M., Jenkinson, M., Woolrich, M. W., Beckmann, C. F., Behrens, T. E. J., Johansen-Berg, H., … Matthews, P. M. (2004). Advances in functional and structural MR image analysis and implementation as FSL. NeuroImage, 23(Suppl 1), S208–S219. 10.1016/j.neuroimage.2004.07.051. 15501092

[ref66] Smith, S. M., & Nichols, T. E. (2009). Threshold-free cluster enhancement: Addressing problems of smoothing, threshold dependence and localisation in cluster inference. NeuroImage, 44(1), 83–98. 10.1016/j.neuroimage.2008.03.061. 18501637

[ref67] Soares, J. M., Marques, P., Alves, V., & Sousa, N. (2013). A hitchhiker's guide to diffusion tensor imaging. Frontiers in Neuroscience, 7, 31. 10.3389/fnins.2013.00031. 23486659 PMC3594764

[ref68] Sprooten, E., Barrett, J., Mckay, D. R., Knowles, E. E., Mathias, S. R., Winkler, A. M., … Glahn, D. C. (2016). A comprehensive tractography study of patients with bipolar disorder and their unaffected siblings. *Human Brain Mapping, **37***(10), 3473–3485. 10.1002/hbm.23253. PMC5496097 27198848

[ref69] Sprooten, E., Sussmann, J. E., Clugston, A., Peel, A., McKirdy, J., Moorhead, T. W. J., … McIntosh, A. M. (2011). White matter integrity in individuals at high genetic risk of bipolar disorder. Biological Psychiatry, 70(4), 350–356. 10.1016/j.biopsych.2011.01.021. 21429475

[ref70] Vai, B., Parenti, L., Bollettini, I., Cara, C., Verga, C., Melloni, E., … Benedetti, F. (2020). Predicting differential diagnosis between bipolar and unipolar depression with multiple kernel learning on multimodal structural neuroimaging. European Neuropsychopharmacology, 34, 28–38. 10.1016/j.euroneuro.2020.03.008. 32238313

[ref71] van Velzen, L. S., Kelly, S., Isaev, D., Aleman, A., Aftanas, L. I., Bauer, J., … Schmaal, L. (2020). White matter disturbances in major depressive disorder: A coordinated analysis across 20 international cohorts in the ENIGMA MDD working group. Molecular Psychiatry, 25(7), 1511–1525. 10.1038/s41380-019-0477-2. 31471575 PMC7055351

[ref72] Versace, A., Almeida, J. R. C., Quevedo, K., Thompson, W. K., Terwilliger, R. A., Hassel, S., … Phillips, M. L. (2010). Right orbitofrontal corticolimbic and left corticocortical white matter connectivity differentiate bipolar and unipolar depression. Biological Psychiatry, 68(6), 560–567. 10.1016/j.biopsych.2010.04.036. 20598288 PMC3743239

[ref73] Vogelbacher, C., Möbius, T. W. D., Sommer, J., Schuster, V., Dannlowski, U., Kircher, T., … Bopp, M. H. A. (2018). The Marburg-Münster Affective Disorders Cohort Study (MACS): A quality assurance protocol for MR neuroimaging data. NeuroImage, 172, 450–460. 10.1016/j.neuroimage.2018.01.079. 29410079

[ref74] Winkler, A. M., Ridgway, G. R., Webster, M. A., Smith, S. M., & Nichols, T. E. (2014). Permutation inference for the general linear model. NeuroImage, 92(100), 381–397. 10.1016/j.neuroimage.2014.01.060. 24530839 PMC4010955

[ref75] Wise, T., Radua, J., Nortje, G., Cleare, A. J., Young, A. H., & Arnone, D. (2016). Voxel-based meta-analytical evidence of structural disconnectivity in major depression and bipolar disorder. Biological Psychiatry, 79(4), 293–302. 10.1016/j.biopsych.2015.03.004. 25891219

[ref76] Wittchen, H.-U., Wunderlich, U., Gruschwitz, S., & Zaudig, M. (1997). SKID I. Strukturiertes Klinisches Interview für DSM-IV. Achse I: Psychische Störungen. Interviewheft und Beurteilungsheft. Eine deutschsprachige, erweiterte Bearb. d. amerikanischen Originalversion des SKID I. Göttingen: Hogrefe.

[ref77] Woolrich, M. W., Jbabdi, S., Patenaude, B., Chappell, M., Makni, S., Behrens, T., … Smith, S. M. (2009). Bayesian analysis of neuroimaging data in FSL. NeuroImage, 45(Suppl 1), S173–S186. 10.1016/j.neuroimage.2008.10.055. 19059349

[ref78] Yang, C., Li, L., Hu, X., Luo, Q., Kuang, W., Lui, S., … Gong, Q. (2019). Psychoradiologic abnormalities of white matter in patients with bipolar disorder: Diffusion tensor imaging studies using tract-based spatial statistics. Journal of Psychiatry & Neuroscience, 44(1), 32–44. 10.1503/jpn.170221. 30565904 PMC6306286

[ref79] Yang, X.-H., Wang, Y., Wang, D.-F., Tian, K., Cheung, E. F. C., Xie, G. R., & Chan, R. C. K. (2017). White matter microstructural abnormalities and their association with anticipatory anhedonia in depression. Psychiatry Research Neuroimaging, 264, 29–34. 10.1016/J.PSCYCHRESNS.2017.04.005. 28437669

[ref80] Young, R. C., Biggs, J. T., Ziegler, V. E., & Meyer, D. A. (1978). A rating scale for mania: Reliability, validity and sensitivity. British Journal of Psychiatry, 133(11), 429–435. 10.1192/bjp.133.5.429. 728692

[ref81] Zanetti, M. V., Jackowski, M. P., Versace, A., Almeida, J. R. C., Hassel, S., Duran, F. L. S., … Phillips, M. L. (2009). State-dependent microstructural white matter changes in bipolar I depression. European Archives of Psychiatry and Clinical Neuroscience, 259(6), 316–328. 10.1007/s00406-009-0002-8. 19255710 PMC2732355

[ref82] Zhu, X., Wang, X., Xiao, J., Liao, J., Zhong, M., Wang, W., & Yao, S. (2012). Evidence of a dissociation pattern in resting-state default mode network connectivity in first-episode, treatment-naive major depression patients. Biological Psychiatry, 71(7), 611–617. 10.1016/J.BIOPSYCH.2011.10.035. 22177602

