# Supplementary material for: Reduced fractional anisotropy in bipolar disorder v. major depressive disorder independent of current symptoms
Source: Psychol Med. 2022 Jul 14;53(10):4592–602. doi: 10.1017/S0033291722001490 (PMC10388324; doi:10.1017/S0033291722001490)
Supplement: Supplementary file 1 [file S0033291722001490sup001.docx]

**Supplemental Material**

**Supplement 1: Choosing participants from the FOR2107 cohort**

The third data freeze (05.01.2021) from the FOR2107 cohort was used with *n*=172 BD, *n*=986 and *n*=1050 HC. Exclusion criteria for participation were age under 18 or above 65 years, the common contraindications against MRI, any history of cardiovascular, neurological and autoimmune diseases, cancer or seizures, severe craniocerebral injury, head trauma or unconsciousness, hypothyroidism without adequate medication, claustrophobia or color blindness. Additionally, choosing participants for this study, participants with a lifetime diagnosis of dysthymia without a depressive episode, schizophrenia, schizoaffective disorder or substance dependence (with the exception of cannabis dependence in bipolar patients) were excluded, as well as participants who lacked general study compliance (*n*=161 BD, *n*=937 MDD, *n*=1017 HC), and participants without DTI, whose DTI image did not survive quality control or who displayed pathological variants in the T1 image (*n*=136 BD, *n*=837 MDD, *n*=945 HC). HC with any life-time psychiatric disorder according to the SCID-I (*n*=825 HC). *N*=136 HC and *n*=136 MDD from this cohort were matched to the remaining *n*=136 BD regard to age, sex and site, using the MatchIt package in R (2020, Version 4.0.1), resulting in the final sample of *N*=408 (*n*=136 per group).

**Supplement 2: Description of the MedIndex**

The Medication Load Index (MedIndex) was calculated by assigning a value between 0 and 2 to each psychopharmacological agent depending on whether that medication was absent (=0), the dose was equal to or lower than the average dose (low=1), or the dose was higher than average (high=2). The average dose was defined by the daily dose intake recommended by the Physician’s-Desk Reference (1). By calculating the sum of all medication scores per participant, the resulting MedIndex reflected both the number and daily dose of all current psychopharmacological medications taken by the participant.

**Supplement 3: Acquisition parameters of MRI data**

Data were acquired using a 3T whole body MRI scanner (Marburg: Tim Trio, Siemens, Erlangen, Germany; Münster: Prisma, Siemens, Erlangen, Germany) using a GRAPPA acceleration factor of 2. Fifty-six axial slices with no gap were measured with an isotropic voxel size of 2.5 x 2.5 x 2.5 mm³ (TE=90ms, TS=7300ms). Five non-diffusion-weighted (DW) images (b=0 s/mm2) and 2x30 DW images with a b-value of 1000 s/mm² were measured. Imaging pulse sequence parameters were standardized across both sites to the extent permitted by each platform.

**Supplement 4: Calculation of DTI metrics**

FA is calculated as the normalized variance of the three eigenvalues and measures the degree of diffusion directionality, with values ranging between 0 (=isotropic diffusion) and 1 (=anisotropic diffusion). MD is defined as the mean of all three eigenvalues and characterizes overall diffusion while RD is calculated as the mean of the second and third eigenvalue and represents motion perpendicular to the tract. AD is equivalent to the primary eigenvalue, reflecting diffusivity along the primary diffusion direction, parallel to the tract (2,3).

**Supplement 5: Effects in RD, MD and AD**

**Analysis 1: HC vs. MDD vs. BD.** A significant main effect of diagnosis across BD, MDD and HC was found on RD (*p_tfce-FWE_*=.008, total k=7933 voxels in ten clusters, peak voxel of largest cluster: x=-16, y=-15, z=35, most affected tracts: forceps minor and bilateral superior longitudinal fasciculus), but not on AD (*p_tfce-FWE_*=.778) or MD (*p_tfce-FWE_*=.087). BD patients had significantly increased RD values compared to MDD patients (*p_tfce-FWE_* =.008, total k=22724 voxels in two clusters, peak voxel of largest cluster: x=-38, y=-42, z=21, most affected tracts: left inferior fronto-occipital fasciculus and bilateral superior longitudinal fasciculus) and HC (*p_tfce-FWE_* =.003, total k=34844 voxels in one cluster, peak voxel: x=-6, y=27, z=11, most affected tracts: forceps minor, left inferior fronto-occipital fasciculus and bilateral superior longitudinal fasciculus), but there was no significant difference between MDD and HC (*p_tfce-FWE_* =.181). The differences between MDD and BD remained significant even after correcting for medication load index, the number of depressive episodes and the number of hospitalizations on top of age, sex, TIV, and scanner differences (Table S3 and Table S4).

**Analysis 2a: ANCOVA with diagnosis, HDRS-scores and their interaction.** Due to non-significant effects in AD and MD in analysis 1, this analysis was calculated for RD only. There was a significant main effect of diagnosis on RD (*p_tfce-FWE_* =.009). Post hoc t-contrast showed significantly increased RD in BD patients compared with MDD patients (*p_tfce-FWE_*=.008, total k=24604 voxels in eight clusters, peak voxel of largest cluster: x=-37, y=-35, z=31), mostly affecting left inferior fronto-occipital fasciculus and bilateral superior longitudinal fasciculus, as in analysis 1 (see Table S4). There was no significant main effect of HDRS-scores (*p_tfce-FWE_*=.127), neither a significant HDRS x diagnosis interaction (*p_tfce-FWE_*=.913). Results remained significant after inclusion of clinical covariates (medication load index, the number of depressive episodes and the number of hospitalizations) in the model (Table S3 and Table S4).

**Analysis 2b: ANCOVA with diagnosis, YMRS-scores and their interaction.** Due to non-significant effects in AD and MD in analysis 1, this analysis was calculated for RD only Again, a significant main effect of diagnosis on RD emerged (*p_tfce-FWE_*=.014), which was further determined by post hoc t-contrast as a reduction in FA in BD patients (*p_tfce-FWE_*=.013, total k=19408 voxels in eight clusters, peak voxel of largest cluster: x=-36, y=-32, z=32). The effect was located predominantly in the SLF bilaterally (see Table S4). Neither the main effect of YMRS-scores (*p_tfce-FWE_*=.949) nor the YMRS x diagnosis interaction were significant (*p_tfce-FWE_*=.794). Again, additionally correcting for clinical covariates did not change the results (Table S3 and Table S4).

**Analysis 3: ANCOVA with diagnosis, mood state, and their interaction.** No significant effects were found in RD (all *p_tfce-FWE_* ≥.065), AD (all *p_tfce-FWE_* ≥.249) and MD (all *p_tfce-FWE_*≥.184).

**Supplement 6: Analysis on differences between BD mood state groups**

To investigate mood state-specific differences within the group of BD patients, a one-factorial ANCOVA with FA (or AD, MD and RD, respectively) as dependent variable and mood state (euthymic vs. depressive vs. manic) as independent variable was conducted. As covariates of no interest, age, sex, TIV, Marburg pre body-coil change (yes/no) and Marburg post body-coil change (yes/no) were included. For this analysis, 14 participants had to be excluded because no information on the current mood state was available for them.

These analyses showed no significant effect of mood state on FA (*p_tfce-FWE_*=.941). Similarly, there was no significant effect of mood state on RD (*p_tfce-FWE_*=.894), MD (*p_tfce-FWE_*=.857), or AD (*p_tfce-FWE_* =.940).

**Supplement 7: Supplementary Figures**

**A.**


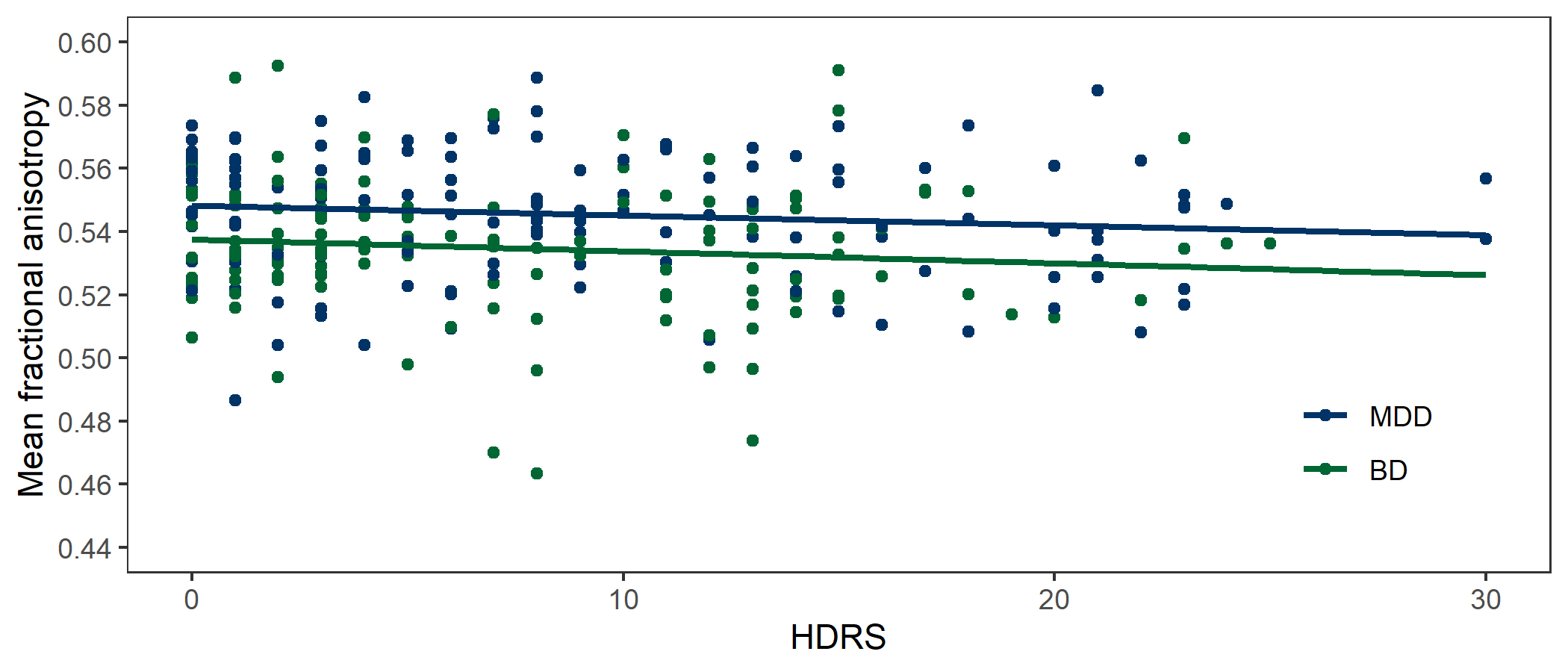


**B.**


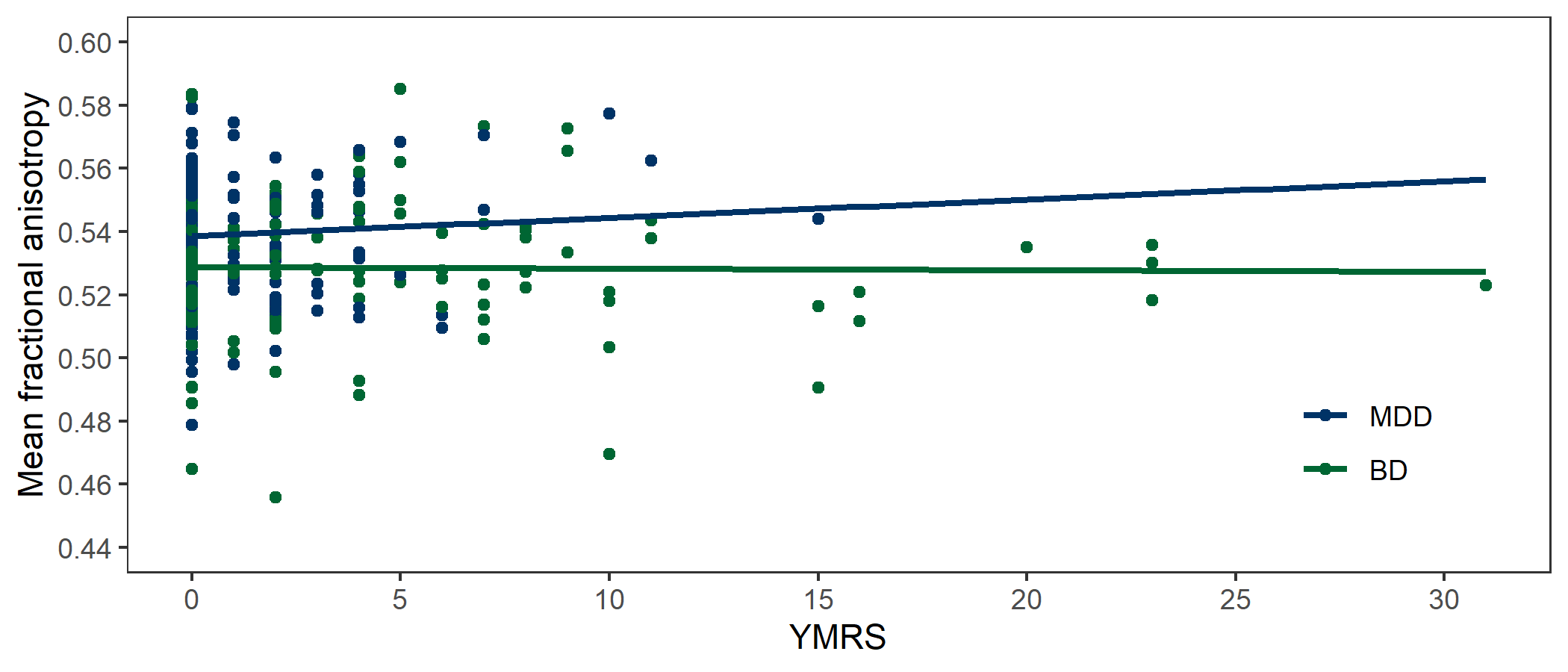


*Figure S1.* Associations between acute symptomatology and mean extracted values of fractional anisotropy (FA) in patients with bipolar disorder (BD) and major depressive disorder (MDD). The mean FA value was obtained from FA values of all the voxels that showed significantly lower FA (*p_tfce-FWE_*<.05) in BD as compared to MDD patients. Continuous lines represent the regression slopes. **A.** Scatter plot depicting the association between scores of the Hamilton Depression Rating Scale (HDRS) and mean extracted FA values. **B.** Scatter plot depicting the association between scores of the Young Mania Rating Scale (YMRS) and mean extracted FA values.

**A. B.**


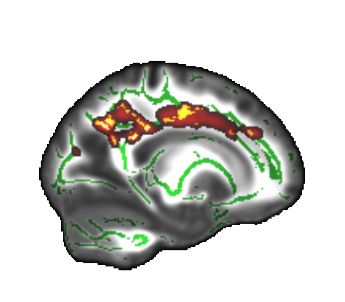

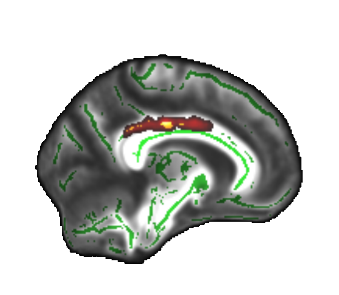

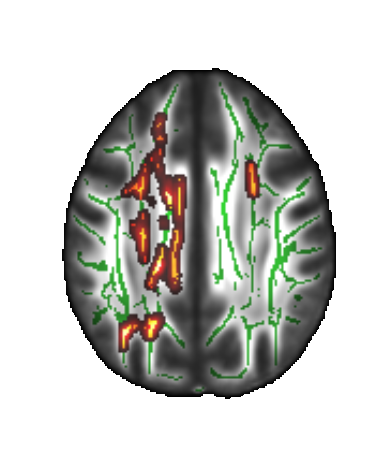

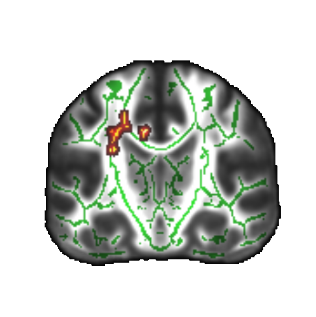


*p* = .036*

*
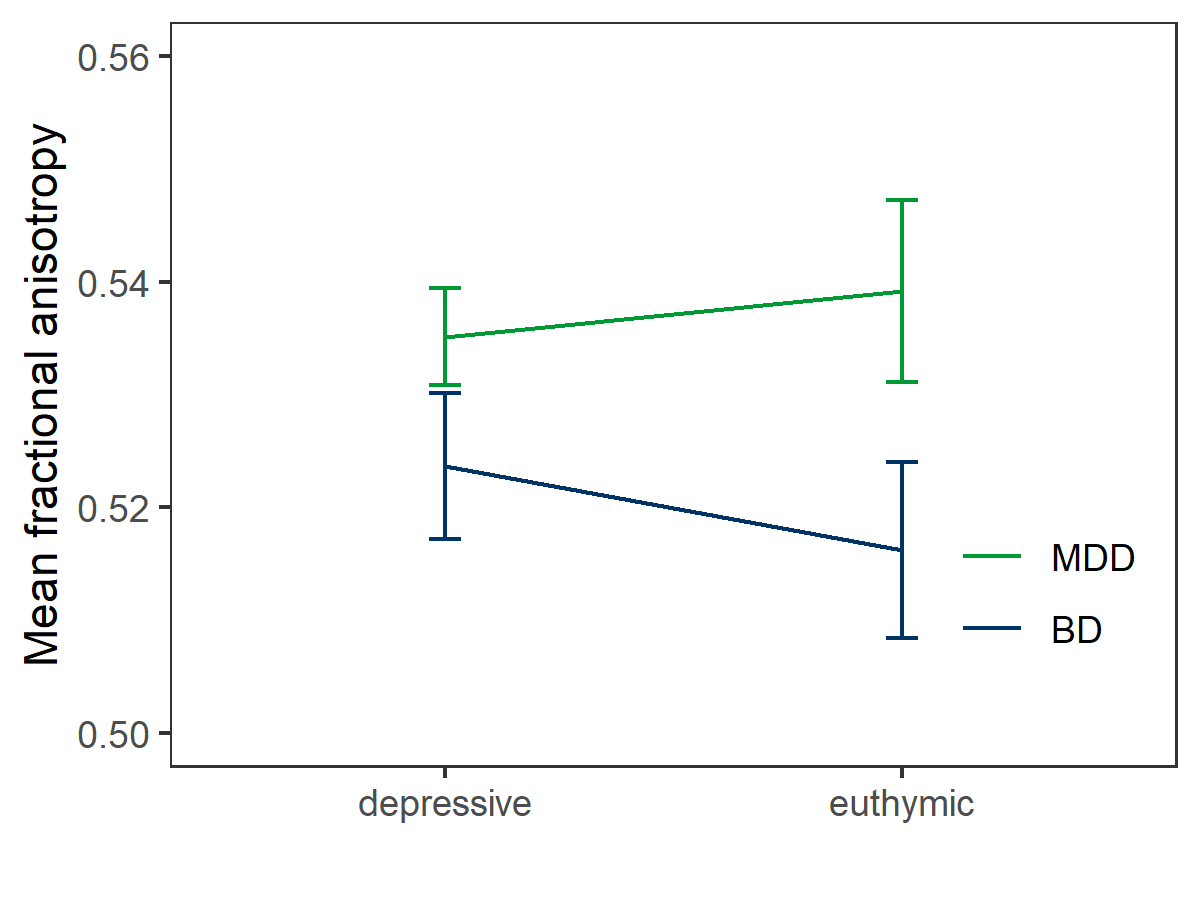
*

*Figure S2*. Reduced fractional anisotropy (FA) in patients with bipolar disorder (BD) compared to patients with major depressive disorder (MDD) in the subsample including only patients in a current euthymic or depressive mood state (ANCOVA with diagnosis, mood state, and their interaction). There was a significant main effect of diagnosis (*p_tfce-FWE_* =.039), for which post hoc *t*-contrast showed a reduction of FA in BD compared to MDD (*p_tfce-FWE_* =.036). There was no main effect of mood state (*p_tfce-FWE_* =.906) nor an interaction of diagnosis and mood state (*p_tfce-FWE_* =.705). **A.** Mean extracted FA values of MDD and BD patients in current depressive and euthymic mood state. The mean FA value was obtained from FA values of all the voxels that showed significantly lower FA (*p_tfce-FWE_*<.05) in BD patients compared to MDD patients in the post hoc *t*-contrast. Error bars represent 95% confidence intervals. **B.** Effect displayed on the FMRIB58 template. Red-yellow areas represent voxels (using FSL’s “fill” command for better visualization), where significantly lower FA (*p_tfce-FWE_*<.05) was detected in patients with BD compared to MDD patients. Top: Saggital views, MNI coordinates: left: x=-19, right: x=-9, Bottom: Coronal (y=-19) and axial views (z=32).

**Supplement 8: Supplementary Tables**

| **Table S1.**  *Medication details of the two patient groups*. | | | | |
| --- | --- | --- | --- | --- |
|  | BD  (*n*=136) | MDD  (*n*=136) | tests for the two patient groups | |
|  |  |  | test statistic | *p*-value |
| Medication load index, M ± SD | 2.57 ± 2.04 | 1.65 ± 1.70 | **-4.041^1^** | **<.001** |
| Antidepressants |  |  |  |  |
| SSNRI | 25 | 42 | **5.723^2^** | **.017** |
| SSRI | 18 | 41 | **11.45^2^** | **.001** |
| NDRI | 6 | 8 | 0.301^2^ | .583 |
| NaSSA | 9 | 15 | 1.645^2^ | .200 |
| NaRI | 1 | 0 | 1.004^2^ | .316 |
| TCA | 7 | 6 | 0.081^2^ | .776 |
| MAOI | 2 | 0 | 2.015^2^ | .156 |
| Agomelatine | 1 | 6 | 3.666^2^ | .056 |
| Lithium | 38 | 5 | **30.08^2^** | **<.001** |
| Antipsychotics | 64 | 28 | **21.29^2^** | **<.001** |
| Benzodiazepines | 0 | 2 | 2.015^2^ | .156 |
| Monotherapy | 49 | 48 | 0.016^2^ | .899 |
| No medication | 22 | 44 | **9.683^2^** | **.002** |
|  |  |  |  |  |
| *Note*: ^1^Two-Sample t-test assuming equal variance, ^2^Pearson χ²-test. Abbreviations: SSNRI, selective serotonin noradrenaline reuptake inhibitor; SSRI, selective serotonin reuptake inhibitor; NDRI, noradrenaline dopamine reuptake inhibitor; NaSSA, noradrenergic and specific serotonergic antidepressant; NaRI, noradrenaline reuptake inhibitor; TCA, tricyclic antidepressants; MAOI, monoamine oxidase inhibitors. | | | | |
| \| **Table S2.**  *Remission status and current affective state of the two patient groups.* \| \| \| \| \| \| \| \| \| \| --- \| --- \| --- \| --- \| --- \| --- \| --- \| --- \| --- \| \| Group \| \| \| Current affective state \| \| \| \| \| Overall \| \| Euthymic \| Depressive \| Manic \| Hypomanic \| Mixed \| \| MDD \| Remission Status \| Acute \| 0 \| 64 \|  \|  \|  \| 64 \| \| Partial remission \| 0 \| 34 \|  \|  \|  \| 34 \| \| Full remission \| 38 \| 0 \|  \|  \|  \| 38 \| \| Overall \| \| 38 \| 98 \|  \|  \|  \| 136 \| \| BD \| Remission Status \| Acute \| 0 \| 29 \| 11 \| 13 \| 1 \| 54 \| \| Partial remission \| 0 \| 24 \| 4 \| 2 \| 3 \| 33 \| \| Full remission \| 39 \| 0 \| 0 \| 0 \| 0 \| 39 \| \| Overall \| \| 39 \| 53 \| 15 \| 15 \| 4 \| 126 \| \| *Note:* Missing data in remission status and/or current affective state for n=10 BD patients. Both variables were determined based on DSM-IV criteria and diagnosis. \| \| \| \| \| \| \| \| \|  \| \| **Table S3.**  *Cluster sizes and MNI coordinates of the peak voxel of all significant clusters, derived with the “cluster” tool implemented in FSL.* \| \| \| \| \| \| \| --- \| --- \| --- \| --- \| --- \| --- \| \| Cluster / contrast \| *p_tfce-FWE_* \| k \| x \| y \| z \| \| Fractional anisotropy \|  \|  \|  \|  \|  \| \| **Main effect of diagnosis: BD vs. MDD vs. HC** \| \| \| \| \| \| \| 1. Cluster \| .018 \| 1194 \| -4 \| 5 \| 24 \| \| 1. Cluster \| .003 \| 817 \| -17 \| -16 \| 34 \| \| 1. Cluster \| .016 \| 167 \| 26 \| 4 \| 32 \| \| 1. Cluster \| .026 \| 144 \| -4 \| 24 \| 15 \| \| 1. Cluster \| .038 \| 55 \| 38 \| -21 \| 31 \| \| 1. Cluster \| .043 \| 17 \| -11 \| 33 \| 8 \| \| 1. Cluster \| .048 \| 13 \| -9 \| 30 \| 0 \| \| 1. Cluster \| .046 \| 12 \| 18 \| 36 \| 2 \| \| 1. Cluster \| .048 \| 12 \| 32 \| 5 \| 32 \| \| 1. Cluster \| .048 \| 9 \| 12 \| 30 \| 11 \| \| 1. Cluster \| .049 \| 5 \| -21 \| -44 \| 28 \| \| 1. Cluster \| .049 \| 2 \| -10 \| 31 \| 12 \| \| 1. Cluster \| .050 \| 1 \| -19 \| -45 \| 26 \| \| **Post-hoc *t*-test: BD < HC** \|  \|  \|  \|  \|  \| \| 1. Cluster \| <.001 \| 38575 \| -14 \| 11 \| 28 \| \| **Post-hoc *t*-test: BD < MDD** \|  \|  \|  \|  \|  \| \| 1. Cluster \| .005 \| 10330 \| -28 \| -17 \| 23 \| \| 1. Cluster \| .011 \| 5712 \| 27 \| 3 \| 30 \| \| 1. Cluster \| .034 \| 1083 \| -19 \| 41 \| 17 \| \| 1. Cluster \| .047 \| 281 \| 24 \| -42 \| 36 \| \| 1. Cluster \| .047 \| 166 \| -24 \| -85 \| -1 \| \| 1. Cluster \| .043 \| 98 \| 38 \| -21 \| 31 \| \| 1. Cluster \| .050 \| 13 \| 30 \| -53 \| 36 \| \| 1. Cluster \| .050 \| 6 \| 36 \| -71 \| 17 \| \| ***t*-test: BD < MDD, additionally corrected for medication load index, the number of depressive episodes and the number of hospitalizations** \| \| \| \| \| \| \| 1. Cluster \| .006 \| 4897 \| -27 \| 4 \| 29 \| \| 1. Cluster \| .020 \| 559 \| 27 \| 4 \| 31 \| \| 1. Cluster \| .049 \| 39 \| 34 \| 20 \| 22 \| \| 1. Cluster \| .050 \| 8 \| 27 \| 15 \| 20 \| \| **Post-hoc *t*-test: BD < MDD, from the ANCOVA with diagnosis, HDRS and their interaction** \| \| \| \| \| \| \| 1. Cluster \| .006 \| 12834 \| -24 \| -24 \| 35 \| \| 1. Cluster \| .007 \| 7109 \| 27 \| 5 \| 31 \| \| 1. Cluster \| .038 \| 311 \| 38 \| -21 \| 31 \| \| 1. Cluster \| .046 \| 193 \| 48 \| -47 \| -4 \| \| 1. Cluster \| .048 \| 115 \| 39 \| -8 \| 26 \| \| **Post-hoc *t*-test: BD < MDD, from the ANCOVA with diagnosis, HDRS and their interaction, additionally corrected for medication load index, the number of depressive episodes and the number of hospitalizations** \| \| \| \| \| \| \| 1. Cluster \| .003 \| 6801 \| -27 \| -6 \| 22 \| \| 1. Cluster \| .012 \| 1493 \| 27 \| 4 \| 31 \| \| 1. Cluster \| .040 \| 749 \| 13 \| -3 \| 2 \| \| 1. Cluster \| .049 \| 22 \| -8 \| -2 \| 33 \| \| 1. Cluster \| .050 \| 7 \| -7 \| -12 \| 34 \| \| **Post-hoc *t*-test: BD < MDD, from the ANCOVA with diagnosis, YMRS and their interaction** \| \| \| \| \| \| \| 1. Cluster \| .008 \| 11085 \| -24 \| -25 \| 33 \| \| 1. Cluster \| .015 \| 4456 \| 27 \| 4 \| 32 \| \| 1. Cluster \| .046 \| 588 \| 17 \| -33 \| 30 \| \| **Post-hoc *t*-test: BD < MDD, from the ANCOVA with diagnosis, YMRS and their interaction, additionally corrected for medication load index, the number of depressive episodes and the number of hospitalizations** \| \| \| \| \| \| \| 1. Cluster \| .015 \| 1559 \| -21 \| -3 \| 34 \| \| 1. Cluster \| .035 \| 267 \| 25 \| 2 \| 34 \| \| 1. Cluster \| .049 \| 27 \| -21 \| -47 \| 37 \| \| 1. Cluster \| .049 \| 17 \| -20 \| -35 \| 32 \| \| 1. Cluster \| .050 \| 14 \| -24 \| -43 \| 35 \| \| **Post-hoc *t*-test: BD < MDD, from the ANCOVA with diagnosis, mood state and their interaction (subsample without manic BD patients)** \| \| \| \| \| \| \| 1. Cluster \| .040 \| 1271 \| -19 \| -33 \| 32 \| \| 1. Cluster \| .036 \| 544 \| -17 \| 3 \| 37 \| \| 1. Cluster \| .042 \| 266 \| -22 \| -23 \| 36 \| \| 1. Cluster \| .043 \| 217 \| -30 \| 3 \| 33 \| \| 1. Cluster \| .036 \| 136 \| 27 \| 6 \| 31 \| \| 1. Cluster \| .046 \| 126 \| -26 \| -11 \| 26 \| \| 1. Cluster \| .048 \| 76 \| -17 \| 33 \| 28 \| \| **Post-hoc *t*-test: BD < MDD, from the ANCOVA with diagnosis, mood state and their interaction (subsample without manic BD patients), additionally corrected for medication load index, the number of depressive episodes and the number of hospitalizations** \| \| \| \| \| \| \| 1. Cluster \| .038 \| 285 \| -26 \| 5 \| 30 \| \| Radial diffusivity \|  \|  \|  \|  \|  \| \| **Main effect of diagnosis: BD vs. MDD vs. HC** \| \| \| \| \| \| \| 1. Cluster \| .008 \| 4716 \| -16 \| -15 \| 35 \| \| 1. Cluster \| .017 \| 2103 \| -27 \| -33 \| 27 \| \| 1. Cluster \| .037 \| 458 \| -26 \| -72 \| 19 \| \| 1. Cluster \| .020 \| 309 \| 11 \| 2 \| 2 \| \| 1. Cluster \| .039 \| 269 \| 22 \| 36 \| 7 \| \| 1. Cluster \| .047 \| 38 \| 34 \| 20 \| 22 \| \| 1. Cluster \| .049 \| 21 \| 12 \| 30 \| -10 \| \| 1. Cluster \| .049 \| 11 \| -25 \| -67 \| 27 \| \| 1. Cluster \| .050 \| 5 \| 34 \| 29 \| 19 \| \| 1. Cluster \| .050 \| 3 \| -16 \| -51 \| 28 \| \| **Post-hoc *t*-test: BD > HC** \|  \|  \|  \|  \|  \| \| 1. Cluster \| .003 \| 34844 \| -6 \| 27 \| 11 \| \| **Post-hoc *t*-test: BD > MDD** \|  \|  \|  \|  \|  \| \| 1. Cluster \| .008 \| 12976 \| -38 \| -42 \| 21 \| \| 1. Cluster \| .019 \| 9748 \| 27 \| 2 \| 31 \| \| ***t*-test: BD > MDD, additionally corrected for medication load index, the number of depressive episodes and the number of hospitalizations** \| \| \| \| \| \| \| 1. Cluster \| .013 \| 9284 \| -26 \| 5 \| 30 \| \| 1. Cluster \| .019 \| 7212 \| 26 \| 0 \| 34 \| \| 1. Cluster \| .047 \| 177 \| -32 \| -62 \| 26 \| \| 1. Cluster \| .047 \| 51 \| -35 \| -3 \| -14 \| \| 1. Cluster \| .049 \| 25 \| -31 \| 2 \| -11 \| \| 1. Cluster \| .050 \| 3 \| 27 \| -67 \| 27 \| \| 1. Cluster \| .050 \| 1 \| -34 \| -1 \| -27 \| \| **Post-hoc *t*-test: BD > MDD, from the ANCOVA with diagnosis, HDRS and their interaction** \| \| \| \| \| \| \| 1. Cluster \| .008 \| 14723 \| -37 \| -35 \| 31 \| \| 1. Cluster \| .016 \| 9638 \| 27 \| 2 \| 31 \| \| 1. Cluster \| .049 \| 93 \| 9 \| 26 \| 22 \| \| 1. Cluster \| .049 \| 62 \| 25 \| -88 \| -1 \| \| 1. Cluster \| .049 \| 52 \| 30 \| -69 \| 13 \| \| 1. Cluster \| .049 \| 17 \| 28 \| -74 \| 6 \| \| 1. Cluster \| .049 \| 10 \| 49 \| -28 \| 7 \| \| 1. Cluster \| .050 \| 9 \| 49 \| -24 \| 4 \| \| **Post-hoc *t*-test: BD > MDD, from the ANCOVA with diagnosis, HDRS and their interaction, additionally corrected for medication load index, the number of depressive episodes and the number of hospitalizations** \| \| \| \| \| \| \| 1. Cluster \| .007 \| 14549 \| -26 \| -12 \| 25 \| \| 1. Cluster \| .012 \| 13244 \| 27 \| 6 \| 27 \| \| **Post-hoc *t*-test: BD > MDD, from the ANCOVA with diagnosis, YMRS and their interaction** \| \| \| \| \| \| \| 1. Cluster \| .013 \| 11837 \| -36 \| -32 \| 32 \| \| 1. Cluster \| .027 \| 7250 \| 16 \| -4 \| 3 \| \| 1. Cluster \| .048 \| 204 \| 19 \| 15 \| 4 \| \| 1. Cluster \| .050 \| 45 \| 32 \| 10 \| -7 \| \| 1. Cluster \| .049 \| 38 \| -13 \| -36 \| 58 \| \| 1. Cluster \| .050 \| 19 \| -12 \| 1 \| 30 \| \| 1. Cluster \| .050 \| 14 \| -10 \| -37 \| 60 \| \| 1. Cluster \| .050 \| 1 \| -13 \| 8 \| 29 \| \| **Post-hoc *t*-test: BD > MDD, from the ANCOVA with diagnosis, YMRS and their interaction, additionally corrected for medication load index, the number of depressive episodes and the number of hospitalizations** \| \| \| \| \| \| \| 1. Cluster \| .022 \| 5515 \| -26 \| -12 \| 26 \| \| 1. Cluster \| .044 \| 481 \| 25 \| 2 \| 34 \| \| 1. Cluster \| .049 \| 225 \| -20 \| -24 \| 38 \| \| 1. Cluster \| .048 \| 132 \| -20 \| -36 \| 33 \| \| 1. Cluster \| .050 \| 72 \| -31 \| -47 \| 16 \| \| 1. Cluster \| .050 \| 67 \| -20 \| -58 \| 31 \| \| 1. Cluster \| .050 \| 66 \| -41 \| -49 \| -1 \| \| 1. Cluster \| .050 \| 57 \| -31 \| -52 \| 24 \| \| 1. Cluster \| .050 \| 49 \| -11 \| -33 \| 31 \| \| 1. Cluster \| .050 \| 40 \| -11 \| -44 \| 27 \| \| 1. Cluster \| .050 \| 33 \| -29 \| -12 \| 44 \| \| 1. Cluster \| .050 \| 27 \| -9 \| -21 \| 32 \| \| 1. Cluster \| .049 \| 24 \| -50 \| -55 \| -2 \| \| 1. Cluster \| .050 \| 24 \| -27 \| -44 \| 20 \| \| 1. Cluster \| .050 \| 17 \| -15 \| -55 \| 24 \| \| 1. Cluster \| .050 \| 9 \| -13 \| -36 \| 58 \| \| 1. Cluster \| .050 \| 9 \| -15 \| -37 \| 37 \| \| 1. Cluster \| .050 \| 7 \| -15 \| -50 \| 27 \| \| 1. Cluster \| .050 \| 4 \| -24 \| -47 \| 32 \| \| 1. Cluster \| .050 \| 4 \| -18 \| -57 \| 34 \| \| 1. Cluster \| .050 \| 3 \| -31 \| -32 \| 44 \| \| 1. Cluster \| .050 \| 3 \| -31 \| -28 \| 44 \| \| 1. Cluster \| .050 \| 2 \| -13 \| -35 \| 37 \| \| *Abbreviations:* BD=Bipolar Disorder, HC=healthy control, k=voxel count, MDD=Major Depressive Disorder, x-y-z=location in three dimensional Montreal Neurological Institute (MNI) space. \| \| \| \| \| \|  \| **Table S4.**  *Anatomical regions comprising the significant effects of the analyses based on the “JHU White-Matter Tractography Atlas”, as implemented in FSL (4–6). The numbers represent the (average) probability of all significant voxels being a member of the different labelled regions within the atlas, calculated with the FSL tool “atlasquery”.* \| \| \| \| \| --- \| --- \| --- \| --- \| \|  \| Probability \| \| \| \| Region \| FA \| \| RD \| \| **Main effect of diagnosis: BD vs. MDD vs. HC** \|  \| \|  \| \| Anterior thalamic radiation, L/R \| 0.01 / 0.08 \| \| 0.1 / 1.59 \| \| Corticospinal tract, L/R \| 0.44 / 2.09 \| \| 1.69 / 0.92 \| \| Cingulum (cingulate gyrus), L/R \| 0.22 / 0.04 \| \| 0.32 / 0.02 \| \| Forceps minor, bilateral \| 2.68 \| \| 4.16 \| \| Forcpes major, bilateral \| - \| \| 0.31 \| \| Inferior fronto-occipital fasciculus, L/R \| - / 0.01 \| \| 0.59 / 0.50 \| \| Inferior longitudinal fasciculus, L \| - \| \| 0.64 \| \| Superior longitudinal fasciculus, L/R \| 0.03 / 1.56 \| \| 4.68 / 2.55 \| \| Superior longitudinal fasciculus (temporal part), L/R \| 0.01 / 0.56 \| \| 2.06 / 0.8 \| \| Uncinate fasciculus, L/R \| - \| \| 0.02 / 0.12 \| \| **Post-hoc *t*-test: BD vs. HC** \| \| \| \| \| Anterior thalamic radiation, L/R \| 0.99 / 1.08 \| 1.75 / 1.15 \| \| \| Corticospinal tract, L/R \| 0.89 / 1.16 \| 1.00 / 1.33 \| \| \| Cingulum (cingulate gyrus), L/R \| 0.47 / 0.03 \| 0.54 / 0.21 \| \| \| Cingulum (hippocampus), L/R \| < 0.01 / 0.01 \| < 0.01 / 0.01 \| \| \| Forceps major, bilateral \| 1.08 \| 0.59 \| \| \| Forceps minor, bilateral \| 2.31 \| 2.30 \| \| \| Inferior fronto-occipital fasciculus, L/R \| 1.93 / 1.65 \| 1.96 / 1.20 \| \| \| Inferior longitudinal fasciculus, L/R \| 1.22 / 0.83 \| 1.29 / 0.64 \| \| \| Superior longitudinal fasciculus, L/R \| 1.67 / 1.82 \| 1.93 / 2.09 \| \| \| Superior longitudinal fasciculus (temporal part), L/R \| 0.73 / 0.66 \| 0.80 / 0.77 \| \| \| Uncinate fasciculus \| 0.56 / 0.25 \| 0.57 / 0.21 \| \| \| **Post-hoc *t*-test: BD vs. MDD** \| \| \| \| \| Anterior thalamic radiation, L/R \| 1.93 / 1.67 \| 1.43 / 1.17 \| \| \| Corticospinal tract, L/R \| 1.32 / 1.13 \| 1.19 / 1.15 \| \| \| Cingulum (cingulate gyrus), L/R \| - 1. / 0.02 \| 0.80 / 0.25 \| \| \| Cingulum (hippocampus), R \| 0.01 \| 0.01 \| \| \| Forceps major, bilateral \| 0.48 \| 0.48 \| \| \| Forceps minor, bilateral \| 0.98 \| 0.89 \| \| \| Inferior fronto-occipital fasiculus, L/R \| 2.65 / 0.76 \| 2.08 / 1.72 \| \| \| Inferior longitudinal fasciculus, L/R \| 1.99 / 0.15 \| 1.52 / 0.68 \| \| \| Superior longitudinal fasciculus, L/R \| 3.44 / 0.65 \| 3.30 / 2.43 \| \| \| Superior longitudinal fasciculus (temporal part), L/R \| 1.72 / 0.20 \| 1.52 / 0.94 \| \| \| Uncinate fasciculus, L/R \| 0.79 / 0.05 \| 0.49 / 0.19 \| \| \| ***t*-test: BD vs. MDD, additionally corrected for medication load index, the number of depressive episodes and the number of hospitalizations** \| \| \| \| \| Anterior thalamic radiation, L/R \| 3.09 / 0.05 \| 1.54 / 1.45 \| \| \| Corticospinal tract, L/R \| 2.55 / 0.01 \| 1.01 / 0.95 \| \| \| Cingulum (cingulate gyrus), L/R \| 1.12 / - \| 0.70 / 0.07 \| \| \| Forceps major, bilateral \| - \| 0.20 \| \| \| Forceps minor, bilateral \| 1.47 \| 1.31 \| \| \| Inferior fronto-occipital fasciculus, L/R \| 2.49 / 0.03 \| 2.11 / 1.96 \| \| \| Inferior longitudinal fasciculus, L/R \| 0.69 / - \| 1.16 / 0.41 \| \| \| Superior longitudinal fasciculus, L/R \| 5.67 / 0.08 \| 3.48 / 1.70 \| \| \| Superior longitudinal fasciculus (temporal part), L/R \| 2.82 / 0.01 \| 1.72 / 0.60 \| \| \| Uncinate fasciculus, L/R \| 0.95 / - \| 0.86 / 0.36 \| \| \| **Post-hoc *t*-test: BD vs. MDD, from the ANCOVA with diagnosis, HDRS and their interaction** \| \| \| \| \| Anterior thalamic radiation, L/R \| 1.86 / 1.65 \| 1.36 / 1.02 \| \| \| Corticospinal tract, L/R \| 1.11 / 1.10 \| 1.11 / 0.99 \| \| \| Cingulum (cingulate gyrus), L/R \| 0.82 / 0.02 \| 0.78 / 0.31 \| \| \| Cingulum (hippocampus), L/R \| <0.01 / 0.01 \| - / 0.01 \| \| \| Forceps major, bilateral \| 0.36 \| 0.65 \| \| \| Forceps minor, bilateral \| 1.11 \| 1.20 \| \| \| Inferior fronto-occipital fasiculus, L/R \| 2.45 / 0.83 \| 2.17 / 1.65 \| \| \| Inferior longitudinal fasciculus, L/R \| 1.77 / 0.19 \| 1.61 / 0.46 \| \| \| Superior longitudinal fasciculus, L/R \| 3.10 / 1.44 \| 3.26 / 2.24 \| \| \| Superior longitudinal fasciculus (temporal part), L/R \| 1.51 / 0.51 \| 1.47 / 0.85 \| \| \| Uncinate fasciculus, L/R \| 0.75 / 0.05 \| 0.66 / 0.17 \| \| \| **Post-hoc *t*-test: BD vs. MDD, from the ANCOVA with diagnosis, HDRS and their interaction, additionally corrected for medication load index, the number of depressive episodes and the number of hospitalizations** \| \| \| \| \| Anterior thalamic radiation, L/R \| 2.14 / 1.77 \| 1.52 / 1.17 \| \| \| Corticospinal tract, L/R \| 1.74 / 0.33 \| 1.00 / 0.80 \| \| \| Cingulum (cingulate gyrus), L \| 0.87 \| 0.56 / 0.30 \| \| \| Cingulum (hippocampus), L/R \| - \| <0.01 / 0.01 \| \| \| Forcpes major, bilareral \| - \| 0.31 \| \| \| Forceps minor, bilateral \| 1.04 \| 1.39 \| \| \| Inferior fronto-occipital fasciculus, L/R \| 2.71 / 1.36 \| 1.87 / 2.02 \| \| \| Inferior longitudinal fasciculus, L \| 1.46 \| 1.13 / 0.74 \| \| \| Superior longitudinal fasciculus, L/R \| 4.48 / 0.17 \| 2.61 / 1.63 \| \| \| Superior longitudinal fasciculus (temporal part), L/R \| 2.21 / 0.05 \| 1.23 / 0.62 \| \| \| Uncinate fasciculus, L/R \| 1.42 / 0.31 \| 0.90 / 0.44 \| \| \| **Post-hoc *t*-test: BD vs. MDD, from the ANCOVA with diagnosis, YMRS and their interaction** \| \| \| \| \| Anterior thalamic radiation, L/R \| 1.56 / 1.67 \| 1.05 / 1.08 \| \| \| Corticospinal tract, L/R \| 1.39 / 1.32 \| 1.21 / 1.25 \| \| \| Cingulum (cingulate gyrus), L/R \| 0.89 / 0.03 \| 0.67 / 0.30 \| \| \| Cingulum (hippocampus), R \| - \| 0.02 \| \| \| Forceps major, bilateral \| 0.45 \| 0.32 \| \| \| Forceps minor, bilateral \| 1.79 \| 1.06 \| \| \| Inferior fronto-occipital fasciculus, L/R \| 2.42 / 0.46 \| 1.91 / 1.18 \| \| \| Inferior longitudinal fasciculus, L/R \| 1.59 / <0.01 \| 1.28 / 0.19 \| \| \| Superior longitudinal fasciculus, L/R \| 3.54 / 0.27 \| 3.77 / 2.14 \| \| \| Superior longitudinal fasciculus (temporal part), L/R \| 1.65 / 0.02 \| 1.68 / 0.65 \| \| \| Uncinate fasciculus, L/R \| 0.65 / 0.06 \| 0.49 / 0.12 \| \| \| **Post-hoc *t*-test: BD vs. MDD, from the ANCOVA with diagnosis, YMRS and their interaction, additionally corrected for medication load index, the number of depressive episodes and the number of hospitalizations** \| \| \| \| \| Anterior thalamic radiation, L/R \| 3.65 / <0.01 \| 2.55 / 0.34 \| \| \| Corticospinal tract, L/R \| 6.16 / 0.02 \| 2.39 / <0.01 \| \| \| Cingulum (cingulate gyrus), L \| 0.07 \| 0.54 \| \| \| Forceps major, bilateral \| - \| <0.01 \| \| \| Forceps minor, bilateral \| - \| 2.10 \| \| \| Inferior fronto-occipital fasciculus, L/R \| 1.91/ - \| 2.72 / 0.31 \| \| \| Inferior longitudinal fasciculus, L \| 0.12 \| 0.53 \| \| \| Superior longitudinal fasciculus, L/R \| 0.98 / 0.12 \| 7.10/<0.01 \| \| \| Superior longitudinal fasciculus (temporal part), L/R \| 0.16 / 0.02 \| 3.25 / <0.01 \| \| \| Uncinate fasciculus, L \| 0.95 \| 1.41 / 0.04 \| \| \| **Post-hoc *t*-test: BD vs. MDD, from the ANCOVA with diagnosis, mood state and their interaction (subsample without manic BD patients)** \| \| \| \| \| Anterior thalamic radiation, L \| 0.25 \| - \| \| \| Corticospinal tract, L \| 4.17 \| - \| \| \| Cingulum (cingulate gyrus), L \| 3.48 \| - \| \| \| Forceps major, bilateral \| 0.23 \| - \| \| \| Forceps minor, bilateral \| 0.05 \| - \| \| \| Inferior fronto-occipital fasciculus, L \| 0.16 \| - \| \| \| Inferior longitudinal fasciculus, L \| 0.23 \| - \| \| \| Superior longitudinal fasciculus, L/R \| 0.57 / 0.01 \| - \| \| \| Superior longitudinal fasciculus (temporal part), L/R \| 0.08 / <0.01 \| - \| \| \| Uncinate fasciculus, L \| 0.01 \| - \| \| \| **Post-hoc *t*-test: BD vs. MDD, from the ANCOVA with diagnosis, mood state and their interaction (subsample without manic BD patients), additionally corrected for medication load index, the number of depressive episodes and the number of hospitalizations** \| \| \| \| \| Anterior thalamic radiation, L \| 0.03 \| - \| \| \| Corticospinal tract, L \| 0.56 \| - \| \| \| Superior longitudinal fasciculus, L \| 0.91 \| - \| \| \| Superior longitudinal fasciculus (temporal part), L \| 0.22 \| - \| \| \| *Abbreviations:* DTI = Diffusion Tensor Imaging, FA= fractional anisotropy, L = left, R = right, RD = radial diffusivity. \| \| \| \| \| \| --- \| --- \| --- \| --- \| --- \| --- \| --- \| --- \| --- \| --- \| --- \| --- \| --- \| --- \| --- \| --- \| --- \| --- \| --- \| --- \| --- \| --- \| --- \| --- \| --- \| --- \| --- \| --- \| --- \| --- \| --- \| --- \| --- \| --- \| --- \| --- \| --- \| --- \| --- \| --- \| --- \| --- \| --- \| --- \| --- \| --- \| --- \| --- \| --- \| --- \| --- \| --- \| --- \| --- \| --- \| --- \| --- \| --- \| --- \| --- \| --- \| --- \| --- \| --- \| --- \| --- \| --- \| --- \| --- \| --- \| --- \| --- \| --- \| --- \| --- \| --- \| --- \| --- \| --- \| --- \| --- \| --- \| --- \| --- \| --- \| --- \| --- \| --- \| --- \| --- \| --- \| --- \| --- \| --- \| --- \| --- \| --- \| --- \| --- \| --- \| --- \| --- \| --- \| --- \| --- \| --- \| --- \| --- \| --- \| --- \| --- \| --- \| --- \| --- \| --- \| --- \| --- \| --- \| --- \| --- \| --- \| --- \| --- \| --- \| --- \| --- \| --- \| --- \| --- \| --- \| --- \| --- \| --- \| --- \| --- \| --- \| --- \| --- \| --- \| --- \| --- \| --- \| --- \| --- \| --- \| --- \| --- \| --- \| --- \| --- \| --- \| --- \| --- \| --- \| --- \| --- \| --- \| --- \| --- \| --- \| --- \| --- \| --- \| --- \| --- \| --- \| --- \| --- \| --- \| --- \| --- \| --- \| --- \| --- \| --- \| --- \| --- \| --- \| --- \| --- \| --- \| --- \| --- \| --- \| --- \| --- \| --- \| --- \| --- \| --- \| --- \| --- \| --- \| --- \| --- \| --- \| --- \| --- \| --- \| --- \| --- \| --- \| --- \| --- \| --- \| --- \| --- \| --- \| --- \| --- \| --- \| --- \| --- \| --- \| --- \| --- \| --- \| --- \| --- \| --- \| --- \| --- \| --- \| --- \| --- \| --- \| --- \| --- \| --- \| --- \| --- \| --- \| --- \| --- \| --- \| --- \| --- \| --- \| --- \| --- \| --- \| --- \| --- \| --- \| --- \| --- \| --- \| --- \| --- \| --- \| --- \| --- \| --- \| --- \| --- \| --- \| --- \| --- \| --- \| --- \| --- \| --- \| --- \| --- \| --- \| --- \| --- \| --- \| --- \| --- \| --- \| --- \| --- \| --- \| --- \| --- \| --- \| --- \| --- \| --- \| --- \| --- \| --- \| --- \| --- \| --- \| --- \| --- \| --- \| --- \| --- \| --- \| --- \| --- \| --- \| --- \| --- \| --- \| --- \| --- \| --- \| --- \| --- \| --- \| --- \| --- \| --- \| --- \| --- \| --- \| --- \| --- \| --- \| --- \| --- \| --- \| --- \| --- \| --- \| --- \| --- \| --- \| --- \| --- \| --- \| --- \| --- \| --- \| --- \| --- \| --- \| --- \| --- \| --- \| --- \| --- \| --- \| --- \| --- \| --- \| --- \| --- \| --- \| --- \| --- \| --- \| --- \| --- \| --- \| --- \| --- \| --- \| --- \| --- \| --- \| --- \| --- \| --- \| --- \| --- \| --- \| --- \| --- \| --- \| --- \| --- \| --- \| --- \| --- \| --- \| --- \| --- \| --- \| --- \| --- \| --- \| --- \| --- \| --- \| --- \| --- \| --- \| --- \| --- \| --- \| --- \| --- \| --- \| --- \| --- \| --- \| --- \| --- \| --- \| --- \| --- \| --- \| --- \| --- \| --- \| --- \| --- \| --- \| --- \| --- \| --- \| --- \| --- \| --- \| --- \| --- \| --- \| --- \| --- \| --- \| --- \| --- \| --- \| --- \| --- \| --- \| --- \| --- \| --- \| --- \| --- \| --- \| --- \| --- \| --- \| --- \| --- \| --- \| --- \| --- \| --- \| --- \| --- \| --- \| --- \| --- \| --- \| --- \| --- \| --- \| --- \| --- \| --- \| --- \| --- \| --- \| --- \| --- \| --- \| --- \| --- \| --- \| --- \| --- \| --- \| --- \| --- \| --- \| --- \| --- \| --- \| --- \| --- \| --- \| --- \| --- \| --- \| --- \| --- \| --- \| --- \| --- \| --- \| --- \| --- \| --- \| --- \| --- \| --- \| --- \| --- \| --- \| --- \| --- \| --- \| --- \| --- \| --- \| --- \| --- \| --- \| --- \| --- \| --- \| --- \| --- \| --- \| --- \| --- \| --- \| --- \| --- \| --- \| --- \| --- \| --- \| --- \| --- \| --- \| --- \| --- \| --- \| --- \| --- \| --- \| --- \| --- \| --- \| --- \| --- \| --- \| --- \| --- \| --- \| --- \| --- \| --- \| --- \| --- \| --- \| --- \| --- \| --- \| --- \| --- \| --- \| --- \| --- \| --- \| --- \| --- \| --- \| --- \| --- \| --- \| --- \| --- \| --- \| --- \| --- \| --- \| --- \| --- \| --- \| --- \| --- \| --- \| --- \| --- \| --- \| --- \| --- \| --- \| --- \| --- \| --- \| --- \| --- \| --- \| --- \| --- \| --- \| --- \| --- \| --- \| --- \| --- \| --- \| --- \| --- \| --- \| --- \| --- \| --- \| --- \| --- \| --- \| --- \| --- \| --- \| --- \| --- \| --- \| --- \| --- \| --- \| --- \| --- \| --- \| --- \| --- \| --- \| --- \| --- \| --- \| --- \| --- \| --- \| --- \| --- \| --- \| --- \| --- \| --- \| --- \| --- \| --- \| --- \| --- \| --- \| --- \| --- \| --- \| --- \| --- \| --- \| --- \| --- \| --- \| --- \| --- \| --- \| --- \| --- \| --- \| --- \| --- \| --- \| --- \| --- \| --- \| --- \| --- \| --- \| --- \| --- \| --- \| --- \| --- \| --- \| --- \| --- \| --- \| --- \| --- \| --- \| --- \| --- \| --- \| --- \| --- \| --- \| --- \| --- \| --- \| --- \| --- \| --- \| --- \| --- \| --- \| --- \| --- \| --- \| --- \| --- \| --- \| --- \| --- \| --- \| --- \| --- \| --- \| --- \| --- \| --- \| --- \| --- \| --- \| --- \| --- \| --- \| --- \| --- \| --- \| --- \| --- \| --- \| --- \| --- \| --- \| --- \| --- \| --- \| --- \| --- \| --- \| --- \| --- \| --- \| --- \| --- \| --- \| --- \| --- \| --- \| --- \| --- \| --- \| --- \| --- \| --- \| --- \| --- \| --- \| --- \| --- \| --- \| --- \| --- \| --- \| --- \| --- \| --- \| --- \| --- \| --- \| --- \| --- \| --- \| --- \| --- \| --- \| --- \| --- \| --- \| --- \| --- \| --- \| --- \| --- \| --- \| --- \| --- \| --- \| --- \| --- \| --- \| --- \| --- \| --- \| --- \| --- \| --- \| --- \| --- \| --- \| --- \| --- \| --- \| --- \| --- \| --- \| --- \| --- \| --- \| --- \| --- \| --- \| --- \| --- \| --- \| --- \| --- \| --- \| --- \| --- \| --- \| --- \| --- \| --- \| --- \| --- \| --- \| --- \| --- \| --- \| --- \| --- \| --- \| --- \| --- \| --- \| --- \| --- \| --- \| --- \| --- \| --- \| --- \| --- \| --- \| --- \| --- \| --- \| --- \| --- \| --- \| --- \| --- \| --- \| --- \| --- \| --- \| --- \| --- \| --- \| --- \| --- \| --- \| --- \| --- \| --- \| --- \| --- \| --- \| --- \| --- \| --- \| --- \| --- \| --- \| --- \| --- \| --- \| --- \| --- \| --- \| --- \| --- \| --- \| --- \| --- \| --- \| --- \| --- \| --- \| --- \| --- \| --- \| --- \| --- \| --- \| --- \| --- \| --- \| --- \| --- \| --- \| --- \| --- \| --- \| --- \| --- \| --- \| --- \| --- \| --- \| --- \| --- \| --- \| --- \| --- \| --- \| --- \| --- \| --- \| --- \| --- \| --- \| --- \| --- \| --- \| --- \| --- \| --- \| --- \| --- \| --- \| --- \| --- \| --- \| --- \| --- \| --- \| --- \| --- \| --- \| --- \| --- \| --- \| --- \| --- \| --- \| --- \| --- \| --- \| --- \| --- \| --- \| --- \| --- \| --- \| --- \| --- \| --- \| --- \| --- \| --- \| --- \| --- \| --- \| --- \| --- \| --- \| --- \| --- \| --- \| --- \| --- \| --- \| --- \| --- \| --- \| --- \| --- \| --- \| --- \| --- \| --- \| --- \| --- \| --- \| --- \| --- \| --- \| --- \| --- \| --- \| --- \| --- \| --- \| --- \| --- \| --- \| --- \| --- \| --- \| --- \| --- \| --- \| --- \| --- \| --- \| --- \| --- \| --- \| --- \| --- \| --- \| --- \| --- \| --- \| --- \| --- \| --- \| --- \| --- \| --- \| --- \| --- \| --- \| --- \| --- \| --- \| --- \| --- \| --- \| --- \| --- \| --- \| --- \| --- \| --- \| --- \| --- \| --- \| --- \| --- \| --- \| --- \| --- \| --- \| --- \| --- \| --- \| --- \| --- \| --- \| --- \| --- \| --- \| --- \| --- \| --- \| --- \| --- \| --- \| --- \| --- \| --- \| --- \| --- \| --- \| --- \| --- \| --- \| --- \| --- \| --- \| --- \| --- \| --- \| --- \| --- \| --- \| --- \| --- \| --- \| --- \| --- \| --- \| --- \| --- \| --- \| --- \| --- \| --- \| --- \| --- \| --- \| --- \| --- \| --- \| --- \| --- \| --- \| --- \| --- \| --- \| --- \| --- \| --- \| --- \| --- \| --- \| --- \| --- \| --- \| --- \| --- \| --- \| --- \| --- \| --- \| --- \| --- \| --- \| --- \| --- \| --- \| --- \| --- \| --- \| --- \| --- \| --- \| --- \| --- \| --- \| --- \| --- \| --- \| --- \| --- \| --- \| --- \| --- \| --- \| --- \| --- \| --- \| --- \| --- \| --- \| --- \| --- \| --- \| --- \| --- \| --- \| --- \| --- \| --- \| --- \| --- \| --- \| --- \| --- \| --- \| --- \| --- \| --- \| --- \| --- \| --- \| --- \| --- \| --- \| --- \| --- \| --- \| --- \| --- \| --- \| --- \| --- \| --- \| --- \| --- \| --- \| --- \| --- \| --- \| --- \| --- \| --- \| --- \| --- \| --- \| --- \| --- \| --- \| --- \| --- \| --- \| --- \| --- \| --- \| --- \| --- \| --- \| --- \| --- \| --- \| --- \| --- \| --- \| --- \| --- \| --- \| --- \| --- \| --- \| --- \| --- \| --- \| --- \| --- \| --- \| --- \| --- \| --- \| --- \| --- \| --- \| --- \| --- \| --- \| --- \| --- \| --- \| --- \| --- \| --- \| --- \| --- \| --- \| --- \| --- \| --- \| --- \| --- \| --- \| --- \| --- \| --- \| --- \| --- \| --- \| --- \| --- \| --- \| --- \| --- \| --- \| --- \| --- \| --- \| --- \| --- \| --- \| --- \| --- \| --- \| --- \| --- \| --- \| --- \| --- \| --- \| --- \| --- \| --- \| --- \| --- \| --- \| --- \| --- \| --- \| --- \| --- \| --- \| --- \| --- \| --- \| --- \| --- \| --- \| --- \| --- \| --- \| --- \| --- \| | | | | |

**References**

1. Reynolds CR (2008): Physician’s Desk Reference. *Encyclopedia of Special Education*. John Wiley & Sons, Inc., pp 1572–1572.

2. Feldman HM, Yeatman JD, Lee ES, Barde LHF, Gaman-Bean S (2010): Diffusion tensor imaging: A review for pediatric researchers and clinicians. *J Dev Behav Pediatr* 31: 346–356.

3. Alexander AL, Lee JE, Lazar M, Field AS (2007): Diffusion Tensor Imaging of the Brain. *Neurotherapeutics* 4: 316–329.

4. Wakana S, Caprihan A, Panzenboeck MM, Fallon JH, Perry M, Gollub RL, *et al.* (2007): Reproducibility of quantitative tractography methods applied to cerebral white matter. *Neuroimage* 36: 630–644.

5. Hua K, Zhang J, Wakana S, Jiang H, Li X, Reich DS, *et al.* (2008): Tract probability maps in stereotaxic spaces: Analyses of white matter anatomy and tract-specific quantification. *Neuroimage* 39: 336–347.

6. Mori S, Wakana S, van Zijl P, Nagae-Poetscher L (2005): *MRI Atlas of Human White Matter*, 1st1. Mori. Amsterdam, The Netherlands: Elsevier.
